# Myotonic dystrophy type 1 embryonic stem cells show decreased myogenic potential, increased CpG methylation at the *DMPK* locus and RNA mis-splicing

**DOI:** 10.1242/bio.058978

**Published:** 2022-01-12

**Authors:** Silvie Franck, Edouard Couvreu De Deckersberg, Jodi L. Bubenik, Christina Markouli, Lise Barbé, Joke Allemeersch, Pierre Hilven, Geoffrey Duqué, Maurice S. Swanson, Alexander Gheldof, Claudia Spits, D. Karen

**Affiliations:** 1 Department Reproduction and Genetics, Vrije Universiteit Brussel, Brussels 1090, Belgium; 2 Department of Molecular Genetics and Microbiology, Center for NeuroGenetics and the Genetics Institute, University of Florida, College of Medicine, Gainesville, FL 32610, USA; 3 Center for Systems and Therapeutics, Gladstone Institutes, San Francisco, 94107 CA, United States; 4 Genomics Core, UZ Leuven, Leuven 3000, Belgium; 5 Center for Medical Genetics, UZ Brussel, Brussels 1090, Belgium

**Keywords:** Myotonic dystrophy type 1, Human embryonic stem cells, Myogenic differentiation, CpG methylation, RNA mis-splicing

## Abstract

Skeletal muscle tissue is severely affected in myotonic dystrophy type 1 (DM1) patients, characterised by muscle weakness, myotonia and muscle immaturity in the most severe congenital form of the disease. Previously, it was not known at what stage during myogenesis the DM1 phenotype appears. In this study we differentiated healthy and DM1 human embryonic stem cells to myoblasts and myotubes and compared their differentiation potential using a comprehensive multi-omics approach. We found myogenesis in DM1 cells to be abnormal with altered myotube generation compared to healthy cells. We did not find differentially expressed genes between DM1 and non-DM1 cell lines within the same developmental stage. However, during differentiation we observed an aberrant inflammatory response and increased CpG methylation upstream of the CTG repeat at the myoblast level and RNA mis-splicing at the myotube stage. We show that early myogenesis modelled in hESC reiterates the early developmental manifestation of DM1.

## Abstract (precis)

**Summary:** Early developmental abnormalities in myotonic dystrophy type 1 are reiterated *in vitro* in myotubes differentiated from human embryonic stem cells that carry the mutation.

## INTRODUCTION

Myotonic Dystrophy type 1 (DM1, OMIM# 160900) is caused by an expanded CTG tract in the 3′ untranslated region (3′ UTR) of the *dystrophia myotonica protein kinase* (*DMPK*) gene that mainly affects muscular and neuronal lineages (Brook et al., 1992; Mahadevan et al., 1992; Udd and Krahe, 2012). The unstable CTG repeat continues to expand over the patients' lifetime resulting in somatic mosaicism (Martorell et al., 1997). The longest CTG expansions have been observed in the most severely affected tissues, including muscle, brain and heart (López Castel et al., 2011). Individuals born with very large CTG expansions can manifest congenital DM1 (CDM), which is the most severe form of the disease (De Antonio et al., 2016; Johnson et al., 2016; Nakamori et al., 2017).

DM1 patients experience muscular symptoms such as muscle weakness, myotonia or loss of muscle strength during disease progression (Bouchard et al., 2015). CDM exhibits features that are not seen in adult or classic DM1 patients, including severe muscle fibre immaturity (Farkas-Bargeton et al., 1988; Nakamori et al., 2017; Sarnat, 2011).

At the molecular level, *DMPK* transcripts containing CUG expansions form toxic RNA foci which sequester splicing factors such as muscleblind-like (MBNL) proteins, while also increasing levels of CUG-binding protein 1 (CELF1), leading to altered RNA splicing events causing DM1-related symptoms (Masuda et al., 2012; Wang et al., 2012). In addition, the hypermethylation of the CpG island upstream of the CTG repeat is seen exclusively in CDM patients and has been suggested to be a biomarker for CDM, arguing in favour of its significant contribution to disease severity (Barbé et al., 2017).

The current knowledge of muscle-specific DM1 mechanisms has been obtained from either mouse models, post-mortem human tissues, patient biopsies or tissue-derived myoblasts. Human embryonic stem cells (hESC) have previously been shown to be a suitable model for DM1 (Seriola et al., 2011; Yanovsky-Dagan et al., 2015; Barbé et al., 2017), although the effect of the DM1 expansion on *in vitro* myogenesis from hESC that carry DM1 has not been reported before.

In this study, we modelled early myoblast and myotube development, starting from hESCs, to investigate differences between healthy and DM1 cells. We detected disease-specific mechanisms at early developmental stages and revealed DM1-specific cellular and molecular pathway deregulation over the time course of early myogenic differentiation.

## RESULTS

### DM1-hESCs differentiate to the myoblast stage, but show a reduced capacity for myotube generation

Six hESC lines were subjected to myogenic differentiation: three non-DM1 cell lines (VUB01, VUB02, VUB06) and three DM1 cell lines (VUB03-DM1, VUB19-DM1, VUB24-DM1), carrying each a differently sized CTG repeat expansion in the *DMPK* locus (Seriola et al., 2011; De Temmerman et al., 2008) (Table 1).

**Table 1.**
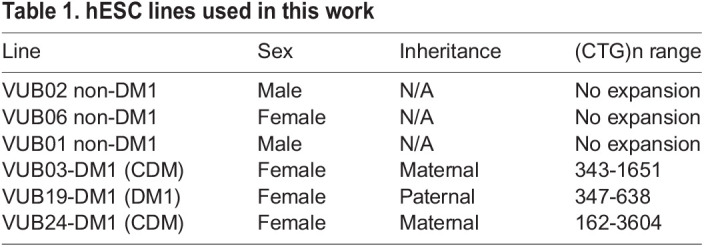
hESC lines used in this work

All six hESC lines were differentiated into myoblasts, selected, and from this stock further differentiated three times to multinucleated myotubes (van der Wal et al., 2017). We attempted to differentiate VUB24-DM1 three times also; however, only one differentiation experiment yielded enough myotube material for the RNAseq experiments. VUB03-DM1 was differentiated three times independently (subline 1, 2, 3) in order to control for differentiation variability. VUB19-DM1 had the smallest repeat size that increased with only 22 repeats from hESC to myotubes (Jonckheere-Terpstra test, *P*=0.009), while the repeat in VUB03-DM1 increased with 151 repeats (Jonckheere-Terpstra, test *P*=0.001). VUB24-DM1 had the largest CTG repeat size and showed the highest size variability (Fig. S1, Table S1).

**Fig. 1.**
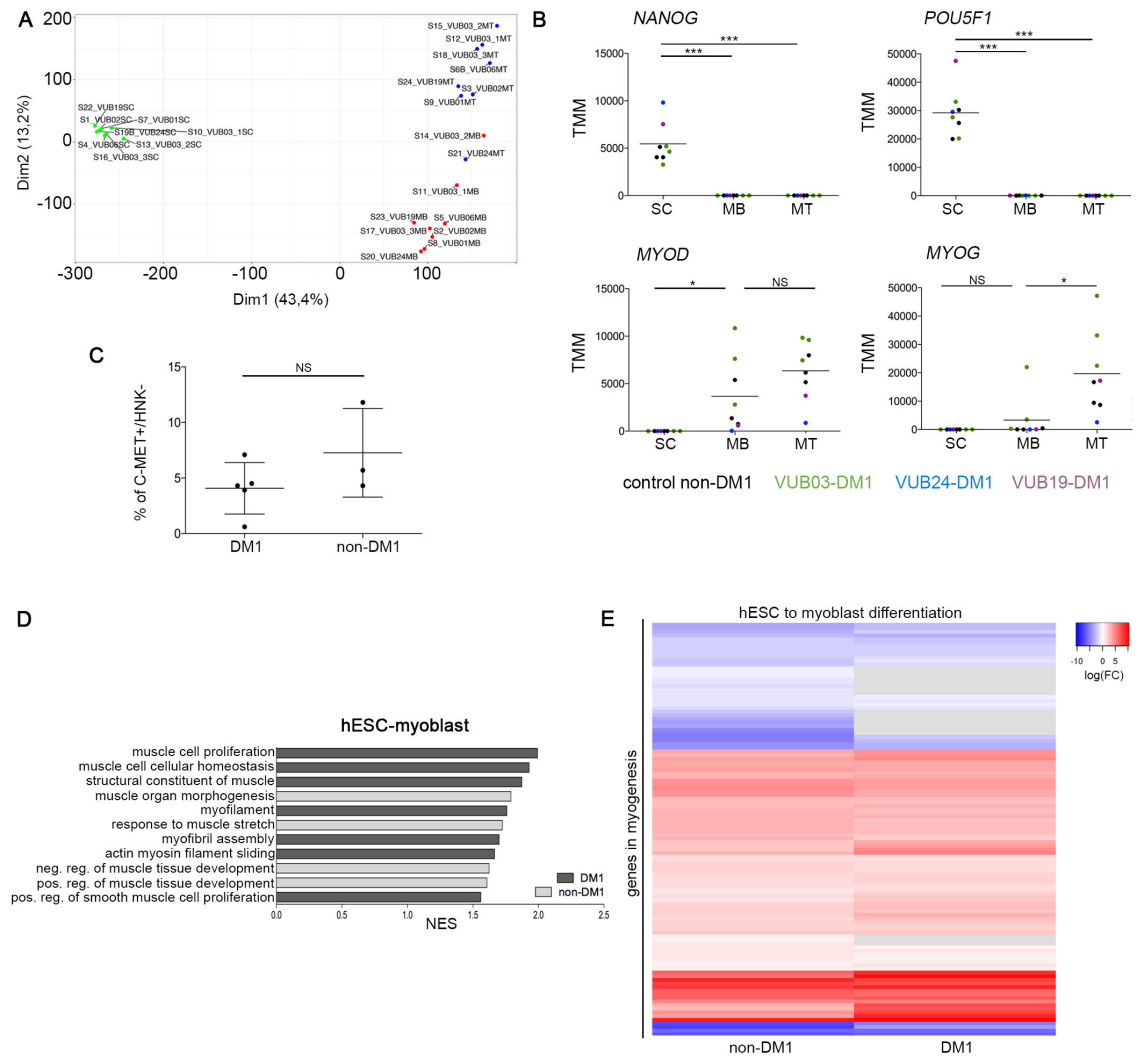
**Myogenic differentiation from hESC to the myoblast stage is equally successful in DM1 and control cell lines.** (A) PCA plot of RNA sequencing results of all lines in the undifferentiated state, the myoblast and the myotube stage. The PCA is based on the results for coding genes with a count per million greater than one in at least two samples. (B) Expression of the undifferentiated state markers *NANOG* and *POU5F1*, and myogenic regulatory factors *MYOD* and *MYOG* over the course of myogenic differentiation, as measured by RNA sequencing. (C) Results of the FACS purification of the myogenic differentiation from hESCs towards myoblasts, using a C-MET+ and HNK1− selection for three non-DM1 lines and three DM1 cell lines (one line in triplicate). Data are shown as means±s.d., *t*-test *P*=0.1935. (D) GO terms for muscle-related gene sets that are significantly enriched in the differential gene expression between hESC and the myoblast stage, using Gene Set Enrichment Analysis. The plot only shows those GO terms that are exclusively enriched for DM1 (dark-grey bars) and non-DM1 (light-grey bars) samples. The full list of 32 commonly enriched gene sets can be found in the Table S2. (E) The heatmap shows the differential gene expression of the genes of the hallmark gene set ‘myogenesis’ of the molecular signatures database, for DM1 and non-DM1 hESC to myoblast differentiation. Genes in grey have an FDR>0.05. The grey bars indicate genes for which the expression level did not change between two developmental stages. The full list of included genes can be found in the Table S4. N/A, not applicable; ND, not determined; NS, not significant; TMM, trimmed mean of M values; SC, human embryonic stem cells; MB, myoblasts; MT, myotubes; FC, fold change; NES, normalized enrichment score.

Transcriptome analysis was carried out by RNA sequencing for all lines at the hESC, myoblast and myotube stage on one sample each. Principle component analysis (PCA) of all samples showed that the myoblast samples clustered apart from myotubes and hESCs, confirming the presence of three different cell identities (Fig. 1A; Fig. S2). In addition, no significant differences based on FDR<0.05 were found when comparing DM1 with non-DM1 samples of the same cell type, including core myogenic regulatory genes *MYOD* and *MYOG*, and loss of markers of undifferentiated state (Fig. 1B).

After the first differentiation step, we found no significant differences in the percentage of C-MET+/HNK− cells in cell cultures by flow cytometry, suggesting that all lines differentiated with similar efficiency (Fig. 1C). We investigated the activation of muscle-related gene sets using gene-set enrichment analysis during the course of differentiation between DM1 and non-DM1 samples. In the differentiation from hESCs to myoblasts, we found 36 muscle-related GO gene sets that were enriched in non-DM1 samples, and 39 that were enriched in DM1 samples, 32 of which were in common (Fig. 1D; full list of pathways in Table S2). The heatmap in Fig. 1E shows the differentially expressed genes of the hallmark gene set ‘myogenesis’ (of the molecular signatures database). We only included those genes that were significantly differentially expressed in the non-DM1 lines (FDR<0.05 in non-DM1 hESC to myoblast differential gene expression). Table S4 shows all the included genes and their log_2_FC and FDR. Overall, both groups show a comparable expression pattern, with both groups similarly inducing muscle-related genes, confirming that DM1 and non-DM1 cell lines undergo the first part of myogenic differentiation with equal efficiency.

**Fig. 2.**
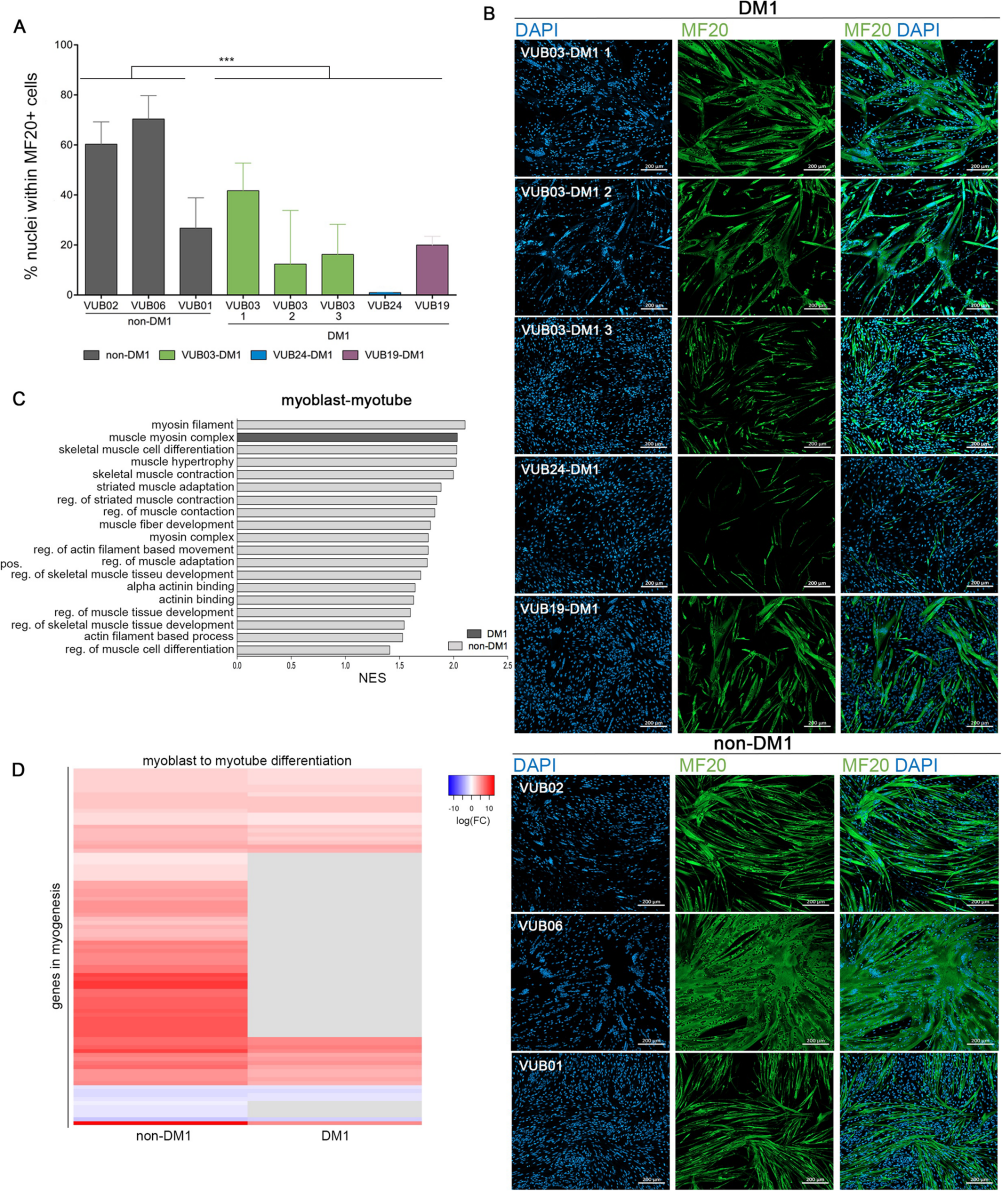
**Myoblasts obtained from DM1-hESC have a decreased ability to progress to the myotube stage.** (A) Percentage of nuclei within a myosin heavy chain (MF20) positive myotube (*n*=3). *** *P*=0.0003, *t*-test. (B) Immunostaining for myosin heavy chain (MF20) for three control non-DM1 cell lines and three DM1 cell lines after myotube differentiation. (C) GO terms for muscle-related gene sets that are significantly enriched in the differential gene expression between the myoblast and myotube stage, using Gene Set Enrichment Analysis. The plot only shows those GO terms that are exclusively enriched for DM1 and non-DM1 samples. The full list of gene sets, including 20 common pathways, can be found in Table S3. (D) The heatmap shows the differential gene expression the hallmark gene set ‘myogenesis’ of the molecular signatures database, for DM1 and non-DM1 myoblast to myotube differentiation. Genes in grey have an FDR>0.05. The grey bars indicate genes for which the expression level did not change between two developmental stages. The full list of included genes can be found in the Table S5. NES, normalized enrichment score; MB, myoblasts; MT, myotubes; FC, fold change.

We then investigated the second stage of the differentiation from myoblasts towards myotubes and found that DM1 lines showed a statistically lower number of MF20+ cells (*t*-test, *P*=0.0003). VUB24-DM1 showed the lowest numbers of positive cells (Fig. 2A,B) and, moreover, VUB24-DM1 myotubes cluster together with the myoblasts in the PCA analysis (Fig. 1B). A gene set enrichment analysis of the differentiation from myoblasts to myotubes, showed 21 GO gene sets that were enriched in DM1 samples, versus 38 that were enriched in non-DM1 samples, of which 20 muscle related GO gene sets were commonly enriched (Fig. 2C; full list in Table S3). Taking into account the genes from the hallmark gene-set ‘myogenesis’, there is a considerable number of genes that are not significantly induced in the DM1 group, which are highly induced in the non-DM1 group (Fig. 2D; Table S5).

### Inflammatory pathways and mTORC1 signalling are differentially activated during the myogenic differentiation of DM1 hESC

**Fig. 3.**
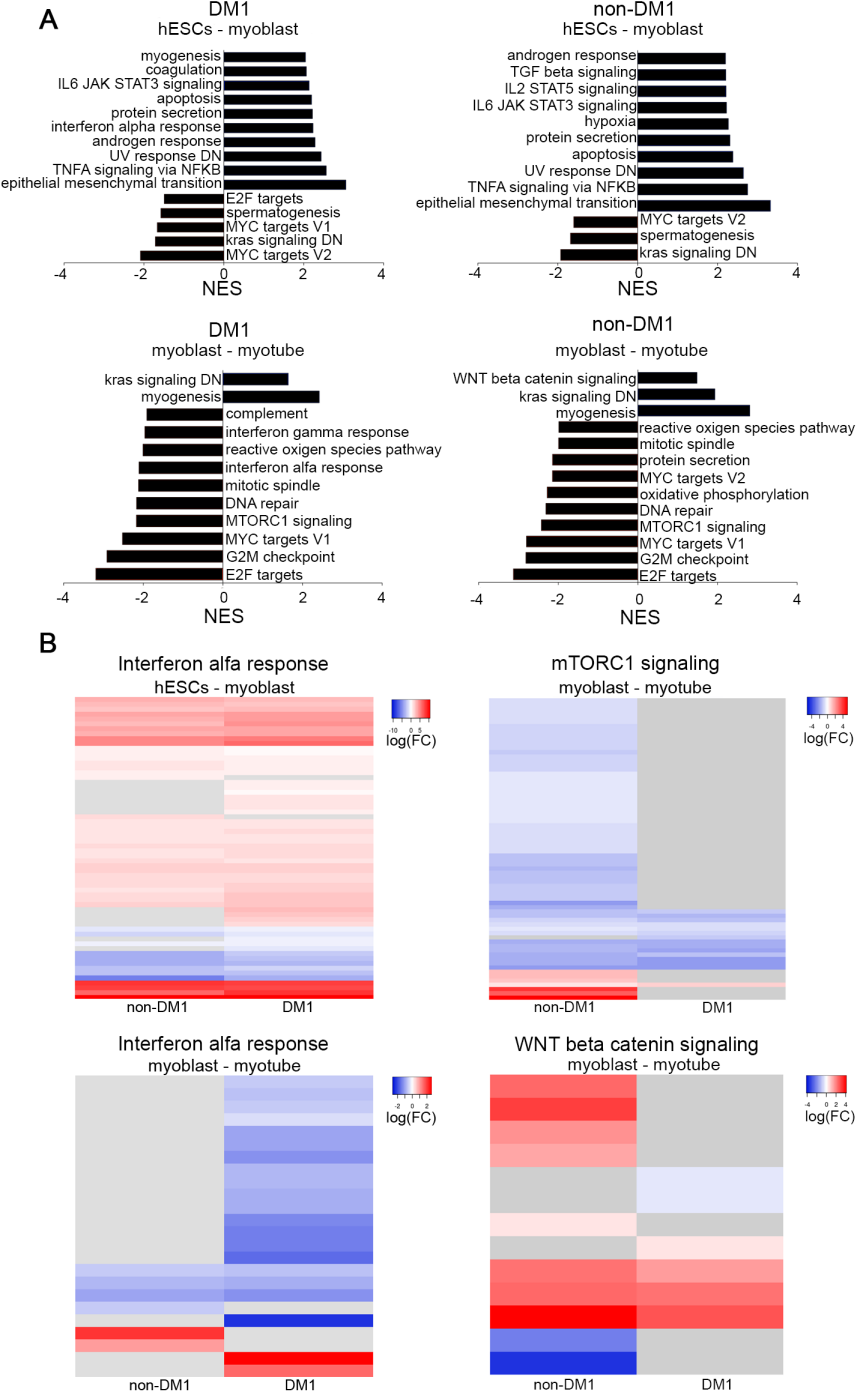
**Pathway analysis in DM1 and non-DM1 samples during myogenic differentiation shows differences in inflammatory response and mTORC1 signalling.** (A) Top ten up- and downregulated pathways. The top panel presents myoblasts compared to hESC, the lower panel represents myotubes compared to myoblasts. (B) Heatmaps representing the log fold change of genes belonging to the interferon alpha response, mTORC1 and canonical WNT signalling. The grey bars indicate genes for which the expression level did not change between two developmental stages. The full list of genes, their log2FC and FDR are listed in Table S6. Grey lines indicate genes with FDR>0.05. NES, normalized enrichment score; FC, fold change.

In order to further explore transcriptional differences in the myogenic differentiation we compared the top positively and negatively enriched Hallmark pathways in differential gene expression from hESCs to myoblasts and from myoblasts towards myotubes (Fig. 3). Interestingly, during the differentiation to myoblasts, we found that IL6-JAK-STAT3 signalling and TNFA signalling via NFKB were positively enriched in both experimental groups (Fig. 3A). On the other hand, the interferon alpha response, belonging to interferon type I, was positively enriched in the differentiation of the DM1 hESC to myoblast only. In the second step of differentiation, the interferon type I pathway appears negatively enriched only in the DM1 cells (Fig. 3A). Remarkably, the canonical WNT pathway was only activated in the non-DM1 myoblast-to-myotube transition. The mTORC1 signalling, an important pathway in myogenesis, was negatively enriched in both groups in the transition from myoblast to myotube (Rion et al., 2019).

We further investigated these pathways by analysing the expression of all genes in each pathway (as listed in the Hallmark gene sets). Fig. 3B and Fig. S3 show the heatmaps of the log_2_FC of all genes in each pathway with an FDR<0.05 in at least one of the groups. Table S6 lists all genes included in the heatmaps and their log_2_FC and FDR. Overall, the majority of genes in the three inflammatory pathways (IL6-JAK-STAT3 signalling, TNFA signalling via NFKB and interferon alpha response) are upregulated in the first part of the differentiation, while in the second part most genes are downregulated, and the only pathway showing differences between the two groups is the interferon alpha response.

In the interferon alpha response pathway, 77% of the genes are upregulated in the DM1 samples and 65% in the non-DM1. Of these upregulated genes, 71% were more strongly induced in the DM1 samples. During the second part of the differentiation, the majority of the interferon alpha response genes were downregulated in the DM1 samples while remaining mostly unchanged in non-DM1. These data support the observation that this pathway was specifically enriched in the DM1 samples during the first part of the differentiation.

With regards to the WNT signalling, the heatmap in Fig. 3B does not reveal pronounced differences in terms of significantly differentially expressed genes between the non-DM1 and DM1 samples, suggesting that the differential enrichment predicted by GSEA is likely not of strong biological relevance.

Finally, the mTORC1 shows striking differences between the two groups. The non-DM1 samples downregulate 87% (62/71) genes in the pathway, suggesting that the mTORC1 signalling is being repressed during the transition from myoblast to myotube. Conversely, the DM1 samples only downregulate 19% (14/71) of the genes, potentially unveiling abnormal activation of the mTORC1 signalling in DM1 myotubes.

In sum, the TNFα signalling via NFKB pathways is equally enriched in both groups. Conversely, the interferon alpha response pathway was only enriched in the DM1 samples, with a stronger up- and downregulation of its genes in the course of differentiation. Further, the mTORC1 signalling showed remarkable differences, with the DM1 cells failing to downregulate this signalling pathway during the progression from myoblast to myotube.

### CpG methylation upstream of the CTG repeat increases over DM1 myogenic differentiation

**Fig. 4.**
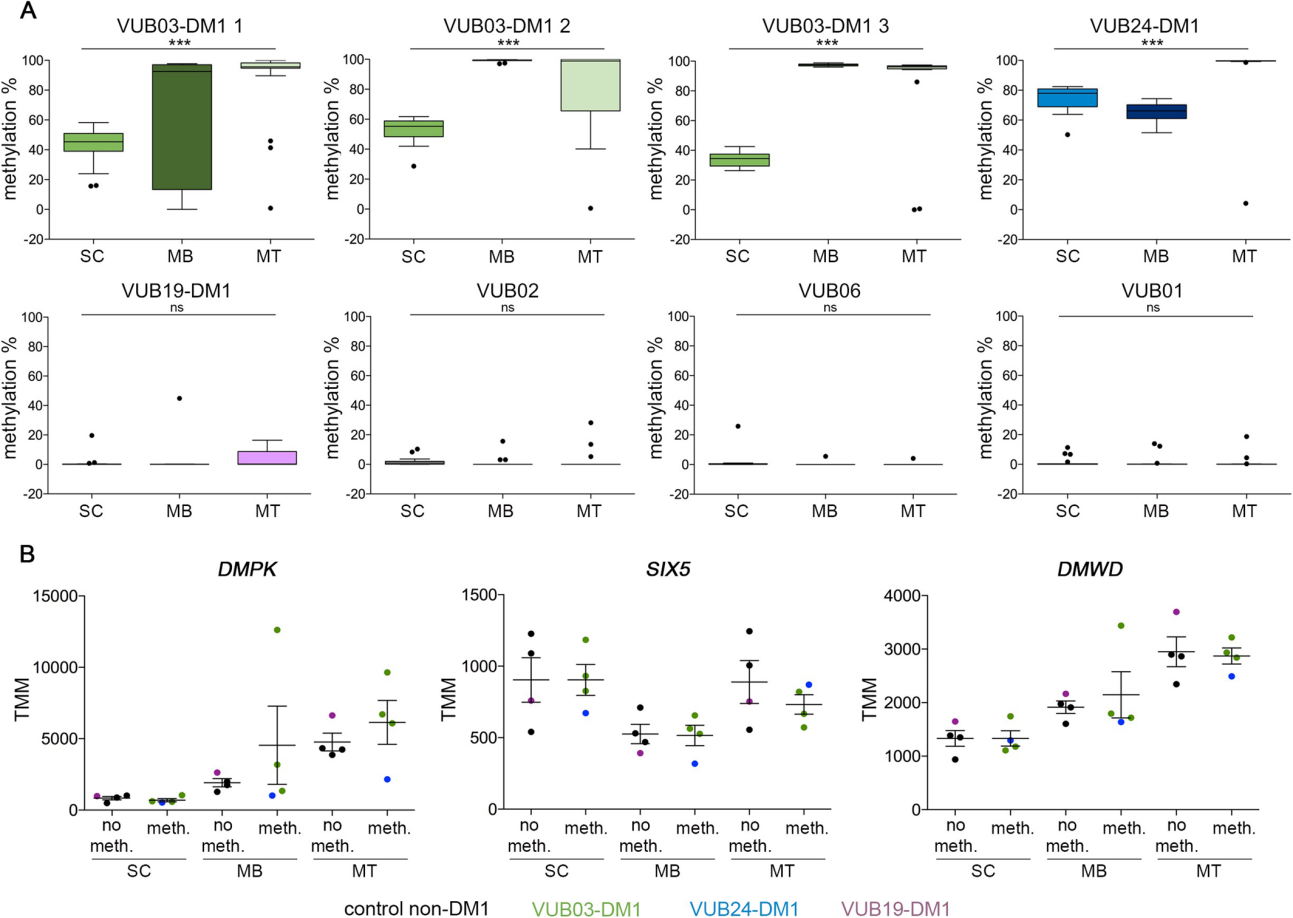
**The upstream CpG methylation in the *DMPK* locus increases over differentiation in DM1 samples but does not affect the expression of *DMPK* and its flanking genes.** (A) Average methylation levels of the CpG sites upstream of the CTG repeat in each sample. The upstream methylation is shown for 23 CpG sites and all epi-alleles were analysed after massive parallel sequencing for three DM1 and three non-DM1 cell lines (one-way ANOVA: VUB03-DM1 1: *P*<0.0001 2: *P*<0.0001 3: *P*<0.0001, VUB24-DM1: *P*<0.0001, VUB19-DM1: *P*=0.2989, VUB02: *P*=0.710, VUB06: *P*=0.326, VUB01: *P*=0.977; *** indicates significant differences). (B) mRNA levels of *DMPK*, *SIX5* and *DMWD* over the course of myogenic differentiation. Samples are grouped according to their CpG methylation status upstream of the CTG repeat. Data are presented as mean±s.e.m. TMM, trimmed mean of M values; SC, hESC; MB, myoblasts; MT, myotubes.

We analysed the methylation status of 23 CpG sites upstream of the CTG repeat, including the CTCF1 region, and 11 downstream CpG sites, spanning the CTCF2 region, in all lines included in this study, at the three stages of differentiation, hESCs, myoblasts and myotubes (Fig. 4).

In the undifferentiated state, two of the three DM1 lines showed methylated alleles for the upstream site, while the third line (VUB19-DM1) showed no methylation, as do the control lines. VUB03-DM1 and VUB24-DM1 carry expansions up to 1600 and 3600 repeats, respectively, while the largest expansions in VUB19-DM1 are only 600 repeats (Table 1; Fig. S1). Conversely, at the downstream site all DM1 hESC lines showed methylation and all controls were fully unmethylated (Fig. S4) and this remained unchanged over time and differentiation.

The methylation levels located upstream of the CTG repeat, however, significantly increased in the course of myogenic differentiation in those lines that already had methylation at their undifferentiated state (Fig. 4, one-way ANOVA, VUB03-DM1 and VUB24-DM1 *P*<0.0001, VUB19-DM1 *P*=0.2989, VUB02 *P*=0.7107, VUB06 *P*=0.3262, VUB01 *P*=0.9774).

Since brain tissue is also affected by DM1 and is known to be hypermethylated in DM1 patients (López Castel et al., 2011), we differentiated the three DM1 lines and two controls towards neuroectoderm as an early developmental stage of nervous tissue.

Neuroectoderm was obtained after 12 days of differentiation as described in Chetty et al. (2013). All DM1 and non-DM1 hESCs differentiated towards neuroectoderm, as indicated by the presence of high levels of the neuroectoderm marker *PAX6* with immunocytochemistry (Fig. S5A) and RT-qPCR (Fig. S5B). As in myogenic cells, a significant increase in methylation of the CpG region upstream of the repeat was observed for VUB03-DM1 1 and VUB24-DM1 (one-way ANOVA, *P*<0.0001, Fig. S6A). The methylation profile of VUB19-DM1 and non-DM1 lines VUB06 and VUB01 remained unmethylated in both cell types (one-way ANOVA, *P*=0.6355, *P*=0.5681, *P*=0.1439, Fig. S6A), while the downstream CpG region remained unchanged (Fig. S6B).

Finally, it has been suggested that an aberrant methylation pattern upstream of the CTG repeat might deregulate the chromatin structure and gene expression at the *DMPK* locus (Furling et al., 2001a; Salvatori et al., 2005; Yanovsky-Dagan et al., 2015). Therefore, we assessed the expression of *DMPK*, *SIX5* and *DMWD* over the full myogenic differentiation process for the samples with and without methylation (VUB03 and VUB24 versus VUB19, VUB01, VUB02 and VUB06, Fig. 4B). The levels of *DMPK* and *DMWD* expression increased over the course of differentiation, and *SIX5* stayed relatively stable, but none significantly differed between methylated and non-methylated samples.

In conclusion, upstream methylation increases upon myogenic as well as neurogenic differentiation in our DM1 samples with the largest repeats and pre-existing methylation, without apparently affecting the expression of *DMPK* and its flanking genes.

### DM1 relevant splicing defects appear in the myotube stage

**Fig. 5.**
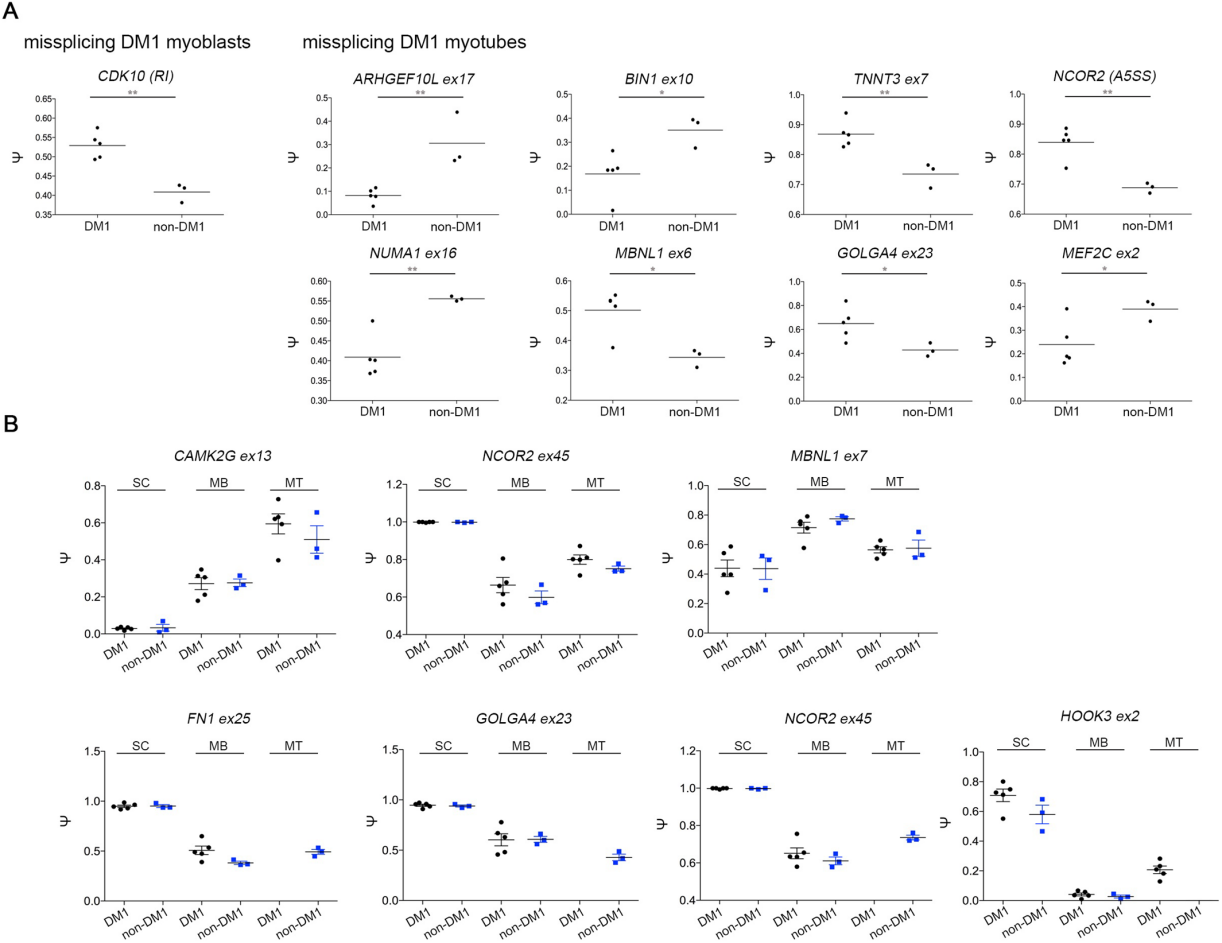
**Expression profile of splicing factors and splicing events in DM1 and non-DM1 hESC, myoblasts and myotubes.** (A) Expression levels of the MBNL-like mRNAs and CELF1 for hESCs, myoblasts and myotubes and for DM1 and non-DM1 samples. (B) Differential splicing events in DM1 hESCs, DM1 myoblasts and DM1 myotubes versus their non-DM1 counterparts. Dot plots were created based on ΔΨ and filtered for number of reads ≥5, FDR ≤0.05 and |ΔΨ|>0.1. (C) Differential splicing events over differentiation from hESCs to myoblasts and from myoblasts to myotubes for DM1 and non-DM1 samples. Dot plots were created based on ΔΨ and cut off values number of reads ≥5, FDR ≤0.05 and |ΔΨ|>0.1. TMM, trimmed mean of M values; SC, hESC; MB, myoblasts; MT, myotubes, A3SS, alternative 3′ splice site; A5SS, alternative 5′ slice site; MXE, mutually exclusive exon; RI, retained intron; SE, skipped exon.

RNA mis-splicing is widespread in DM1 and is primarily driven by the sequestration of MBNL proteins on the CUG expanded DMPK transcripts and an increase in CELF1 levels. We first compared the RNA levels for these factors across myogenic differentiation in both non-DM1 and DM1 cell lines. There were no significant differences based on disease status, however, the levels were modulated by differentiation state (Fig. 5A).

We analysed RNA alternative splicing switches during myogenic differentiation and compared these profiles between the non-DM1 and DM1 lines.

First, we analysed splicing changes in response to differentiation. Unsurprisingly there were many developmentally regulated alternative splicing switches with similar numbers of splicing changes in both the non-DM1 and DM1 cell lines (Fig. 5C). The transition from hESC to myoblast displayed 2967 events in DM1 and 2874 events in non-DM1 lines, while the myoblast to myotube switch resulted in 1510 and 1640 events, respectively. We then examined differences between disease and control samples at each of the developmental points, and found 231 differential splicing events in hESCs, 239 in myoblasts and 261 in myotubes (Fig. 5B).

**Fig. 6.**
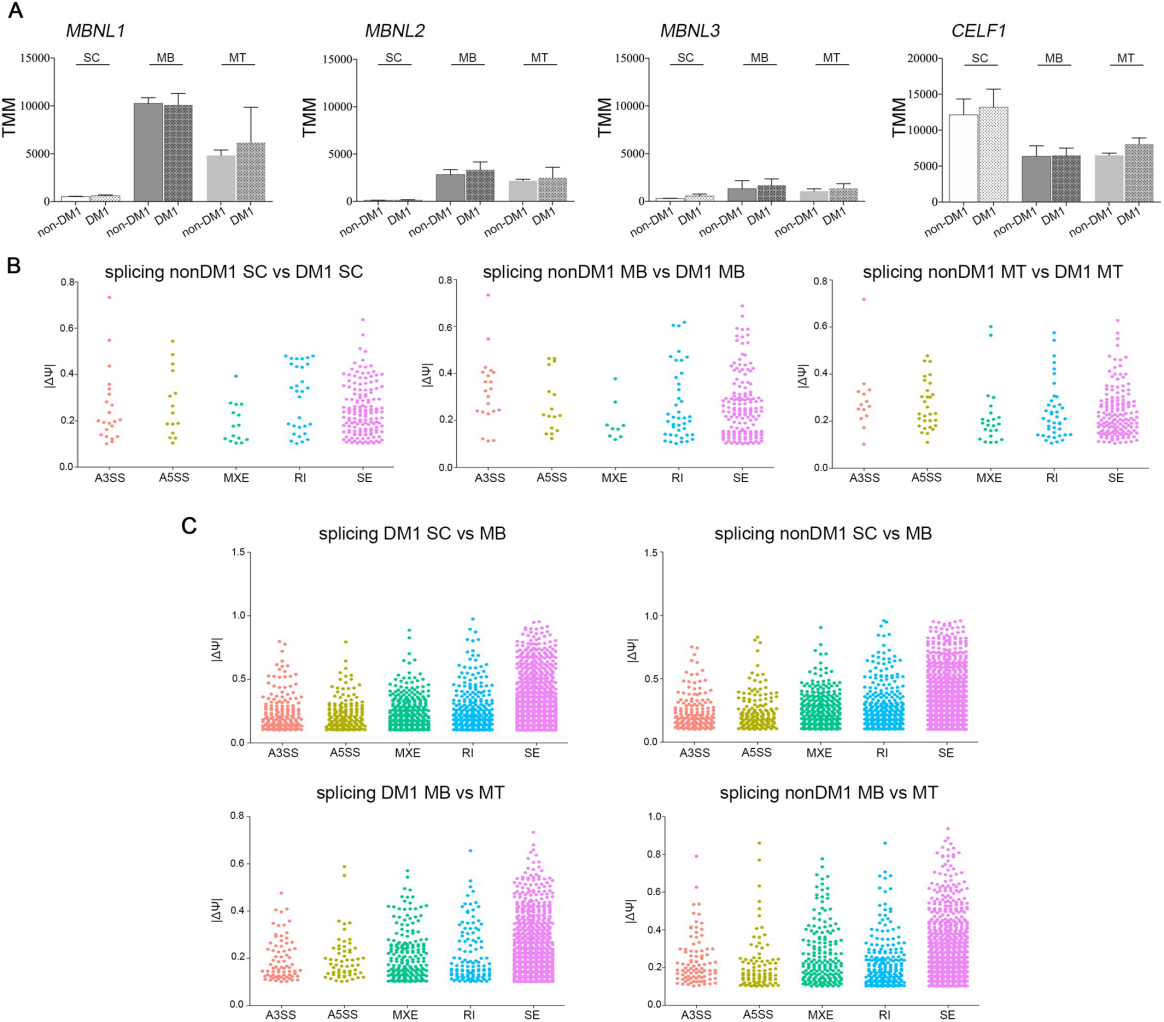
**Specific mis-splicing in DM1 and non-DM1 samples.** (A) Mis-splicing in the myoblasts stage and myotubes stage when comparing DM1 and non-DM1 samples. * and ** indicate significantly different results as calculated with *t*-test. (B) Splice variants over differentiation from hESCs to myoblasts and further towards myotubes. No data means no splice variants detected. SC, hESC; MB, myoblasts; MT, myotubes.

Several previous studies have examined DM1 mis-splicing events in various contexts (Jenquin et al., 2019; Nutter et al., 2019; Thomas et al., 2017; Wagner et al., 2016). Within our dataset, previously identified DM1-associated events were predominantly found at the myotube stage and examples include *ARHGEF10L*, *BIN1*, *TNNT3*, *NCOR2*, *NUMA1*, *MBNL1*, *GOLGA4*, and *MEF2C* (Fig. 6A) though *CDK10* and *MACF1* were already present at the myoblast stage. In addition, transcripts for *INSR* and *SEMA6C* started to appear in non-DM1 samples but were absent in DM1 samples at this stage. The difference in repeat length across our three DM1 hESC lines introduces additional variability into the splicing data, and when we perform the same analysis with the VUB03 sublines only, more DM-1 associated events are detected. These events are trending similarly in the complete set but are unable to pass the filter cut-off due to larger variance in the |Ψ| or FDR values. In addition to these previously identified DM1-associated events, we observed that *PARP2*, *SLC3A2*, *METTL2B* and *CPNE1* all contained alternative splicing events that already occur in hESCs but the potential impact of these differences on disease or differentiation remains to be elucidated. Additional significant DM1-specific mis-splicing events in *LDB3*, *MACF1*, *NDUFV3*, *SLAIN2* and *SORBS1* appeared in the myotube stage.

Over the full time course of myogenic differentiation, we were able to follow a few DM1-specific skipped exon transcripts. Transcripts for *CAMK2G*, *NCOR2* and *MBNL1* seem to follow the same trend during differentiation in DM1 and non-DM1 samples (Fig. 6B). In contrast, *FN1ex25*, *GOLGA4ex23*, *NCOR2ex45* and *HOOK3ex2* follow the same trend from ESCs to myoblasts but show a different behaviour between DM1 and non-DM1 samples at the myotube stage (Fig. 6B). For *HOOK3ex2* the splice isoform persists in the DM1 myotubes while disappearing in the non-DM1 myotubes, while the reverse holds for *FN1ex25*, *GOLGA4ex23*, and *NCOR2ex45*, confirming the data obtained comparing mis-splicing between DM1 and non-DM1 within cell types (Fig. 6A), i.e. that splicing differences between DM1 and non-DM1 samples start to appear at the myotube stage. Tables S7–14 show the comparisons between non-DM1 and DM1 samples, as well as between different differentiation stages. The data were filtered with thresholds averaging five reads or greater, 10% change in splicing, and FDR<0.05.

## DISCUSSION

In this report we investigated the first steps in the DM1 disease process using hESCs that naturally carry the DM1 mutation with a focus on early myogenic differentiation. We found that while both DM1 and non-DM1 lines equally differentiated to the myoblast stage, DM1 cells less efficiently underwent further maturation to myotubes. This observation may reflect the immature muscle phenotype seen in DM1 patients (Nakamori et al., 2017).

While some reports show that cells with a repeat size in the CDM range do not always display an impaired differentiation potential (Loro et al., 2010; Rizzo et al., 2018) and that removal of CTG repeats by CRISPR/Cas9 does not change the capacity of the cells to form myotubes (Provenzano et al., 2017), others report an increased fusion capacity in repeat-deleted cells (van Agtmaal et al., 2017). In our hands, expanded repeats retain their unstable character during myogenic differentiation but we were not able to demonstrate a correlation between large CTG repeats and differentiation potential.

Another aspect we investigated is the activation of specific inflammatory pathways in the course of differentiation. Proinflammatory factors like IL6 and TNFA are necessary for the proliferation of muscle progenitor cells while suppressing further muscle differentiation (Otis et al., 2014), which is in line with these pathways being positively enriched in the first part of differentiation, and negatively enriched in the second part, both for DM1 and non-DM1 hESC lines. Conversely, abnormal activation of the IL6-STAT3 signalling pathway via the activation of NFKB has been proposed as an underlying cause of the immature muscle state observed in CDM (Nakamori et al., 2017). These authors proposed that hypermethylation upstream of the CTG repeat deregulates *DMPK* transcription resulting in a higher level of toxic RNA, which leads to activation of the IL6 pathway. Recently, activation of the interferon type 1 (IFN1) pathway was also shown to be involved in impaired myogenesis and was suggested to be activated by the toxic RNA foci (Rizzo et al., 2018). In our model, we found that IL6-JAK-STAT3 signalling and TNFA signalling via NFKB were positively enriched in both experimental groups, albeit that the IL6-JAK-STAT3 signalling may have been more strongly upregulated in the control group, while the interferon alpha response was exclusively enriched in the DM1 cells. Given that the IFN1 response has been associated with an inhibition of myogenesis (Rizzo et al., 2018), its strong induction in the myoblast stage may be partially responsible for the poorer myotube formation in the DM1 cell lines. A sustained proinflammation signal in the beginning of myotube formation may hamper further myogenic differentiation (Dort et al., 2019), resulting in the observed decreased efficiency of the DM1 cells to generate myotubes.

Our model potentially also confirmed the involvement of mTORC1 in myogenesis (Rion et al., 2019) as well as in DM1 muscle pathology. Brockhoff et al. (2017) demonstrated in a DM1 mouse model that mTORC1 signalling remained active in mutant mice subjected to starvation, while mTORC1 signalling becomes inactive in normal mice (Brockhoff et al., 2017). In our model, while mTORC1 signalling was comparably active in myoblasts (data not shown), it was clearly downregulated in non-DM1 myoblasts differentiating towards myotubes, while this was not the case in DM1 myotubes. Whether mTORC1 is activated (Brockhoff et al., 2017) or on the contrary inhibited (Beffy et al., 2010; Denis et al., 2013) in DM1 remains a topic of controversy. However, further functional experiments in our model would contribute to help unravel the exact role of mTORC1 signalling in DM1 myogenesis.

Muscle tissue from DM1 patients and fetuses, and isolated DM1 myoblasts and myotubes, are known to display high levels of methylation on the CpG island upstream of the CTG repeat (López Castel et al., 2011; Nakamori et al., 2017; Rizzo et al., 2018) and muscle immaturity itself has suggested to be linked to hypermethylation of this site (Nakamori et al., 2017). In our model we showed that the CpG methylation increased over myogenic differentiation, especially in those hESC lines that already showed hypermethylated in the undifferentiated state and which displayed the largest repeat sizes, confirming previous reports indicating that upstream methylation is associated with large CTG repeats and congenital DM1 (Barbé et al., 2017; Yanovsky-Dagan et al., 2015). In line with the published results on CDM1 fetal and newborn myoblasts, we found that this increased methylation is already established at the myoblast stage and even further increases during differentiation to the myotube stage.

It has been suggested that hypermethylation of the *DMPK* flanking CpG regions affects the expression levels of flanking genes, *DMWD* upstream and *SIX5* downstream (Brouwer et al., 2013; Eriksson et al., 2001; Yanovsky-Dagan et al., 2015). The expression of *DMPK* has been suggested to be essential in myogenesis and further in muscle function (Carrell et al., 2016). In our study, we could not find a significant difference in expression levels of *DMPK, SIX5* and *DMWD* between lines with hypo- and hypermethylation, nor a correlation between the methylation and the differentiation capacity. This suggests that the methylation status of this region is not controlling the expression of these genes at this stage of myogenesis and is not a likely explanation for the observed differentiation differences between DM1 and non-DM1 cells.

During myogenic development, mRNA alternative splicing transitions are essential for a proper tissue function and muscle physiology (Brinegar et al., 2017). Alternative splicing is misregulated in DM1 and even more so in CDM1 patients leading to developmentally inappropriate RNA isoforms, eventually causing multisystemic DM1 symptoms (Nakamori et al., 2017; Thomas et al., 2017). In a DM1 mouse knock-in model, in which CTG expansions were inserted into the 3′ UTR of the mouse Dmpk gene, Nutter et al. (2019) demonstrated that the expanded CTG repeat has more severe effects on muscle progenitor stages such as myoblasts and myotubes because of the higher expression of Dmpk at those stages and therefore higher capacity to sequester proteins leading to significant RNA mis-splicing (Nutter et al., 2019). Wagner et al. (2016) proposed 46 aberrant splicing events related to DM1 that could serve as DM1 biomarkers (Wagner et al., 2016). We found the majority of the alternative splicing events proposed by Wagner et al. (2016) in our dataset, of which for example *SORBS1*, *NUMA1*, *MBNL1*, *GOLGA4* and *MEF2C* were significantly different to non-DM1 samples at the myotube stage. This indicates that splicing differences only appear from the myotube stage on and therefore mis-splicing might not be the cause of aberrant myogenesis in earlier stages. In addition, we and others showed that *DMPK* levels increase from the myoblast to the myotube stage (Furling et al., 2001b; Wang et al., 2019), likely increasing the mutated, toxic *DMPK* transcript load which could explain why alternative splicing abnormalities become significantly apparent only at the myotube stage.

Mis-splicing of *BIN1* has been linked to T tubule alterations and muscle weakness (Fugier et al., 2011) and was particularly mis-spliced in the poorly differentiating VUB24-DM1 line. This may indicate that a developmental switch to the correct *BIN1* isoform is not only essential postnatally and in adulthood as suggested by others (Fugier et al., 2011) but during early myogenesis as well. More recently, the precise *BIN1* isoform regulation has been shown to be essential for normal muscle development, maturation and function (Cowling et al., 2017; Prokic et al., 2020).

Mis-splicing of *CLCN1*, a skeletal muscle specific chloride channel which causes myotonia (Pistoni et al., 2010), was not identified since *CLCN1* is not yet expressed in these early developmental stages. Interestingly, the insulin receptor (*INSR*) and the sarcoplasmic/endoplasmic reticulum calcium ATPase 1 (*SERCA1*) transcripts which were also suggested to be mis-spliced in DM1 skeletal muscle myotubes (Provenzano et al., 2017), started to appear in myotubes of non-DM1 samples, but failed to do so in non-DM1 samples. Other mis-splicing events such as in *TNNT3*, *MYOM1*, *TNN*, *TRIM55* and *MYO5A* were also observed. Several of these transcripts were linked to actin cytoskeleton and function (Koebis et al., 2011).

In summary, our findings show that modelling myogenesis using DM1 hESCs recapitulates a number of key cellular and genetic phenotypes that have been previously associated to DM1 pathogenesis. DM1 hESCs have less effective induction of myotube formation than control hESCs, and at the transcriptome level, the model recapitulates previously published observations showing an elevated interferon type I response as an intrinsic feature specific to DM1 myogenesis. We found that myogenic differentiation of DM1 hESC increased already present CpG hypermethylation in the region upstream of the CTG repeat. Finally, we demonstrated that misregulated alternative splicing events start to occur from the myotube stage on, later during myogenic differentiation. We showed that our *in vitro* model is interesting and relevant to study early DM1 pathogenesis and to further unravel abnormal myogenesis.

## MATERIALS AND METHODS

### Cell culture and skeletal myogenic progenitor differentiation

The VUB hESC lines were derived and characterized in our laboratory (Mateizel et al., 2006) and are registered in the EU hESC registry (https://hpscreg.eu/). hESC were cultured at 37°C in 5% CO_2_ and atmospheric O_2_ conditions, on 10 µg/ml laminin-521 (LN521; Biolamina) and in NutriStem^®^ hESC XF medium (Biological Industries), supplemented with 100 U/ml penicillin/streptomycin (Pen/Strep; Thermo Fisher Scientific). Cell passaging was performed using 1x TrypLE™ Express (Thermo Fisher Scientific).

Skeletal myogenic progenitor differentiation of all hESCs lines was performed according to protocols described in van der Wal et al. (2017), with only few adjustments. Briefly, a total of 50,000 cells were plated on a 1.9 cm^2^ dish, coated with 10 µg/ml laminin-521 (LN521; Biolamina). The next day, differentiation was induced by using 10 µM CHIR99021 (Axon MEDCHEM), in myogenic differentiation medium composed of DMEM-F12, 1x ITS-X, 100x Penicillin/Streptomycin/L-Glutamine (all from Thermo Fisher Scientific) (adapted from van der Wal et al. 2017) for 2 days. Subsequently, the CHIR99021 component in the myogenic differentiation medium was replaced by 20 ng/ml FGF2 (Prepotech) for the following 14 days. Myogenic differentiation medium without supplement was used for the last days of the 35 days during differentiation protocol. Medium was refreshed daily.

Following 35 days of differentiation, purification of myogenic progenitors from a mixed cell population was performed using FACS sorting as described in van der Wal et al. (2017). Briefly, cells were harvested with 1x TrypLE™ Express (Thermo Fisher Scientific) and filtered using a 40 µM FACS strainer. Subsequently, the cell suspension was incubated with the appropriate fluorochrome-labelled antibodies (Table S8) as mentioned by van der Wal et al. (2017). Labelled cells were sorted through a FACSAria (BD Biosciences) and collected in myogenic progenitor proliferation medium composed of DMEM high glucose, 10% fetal bovine serum, 100x Penicillin/Streptomycin/L-Glutamine, 1x RevitaCell supplement (all from Thermo Fisher Scientific) and 100 ng/ml FGF2 (Prepotech) (adopted from van der Wal et al. 2017). Captured cells were subsequently plated on a 10 µg/ml laminin-521 (LN521; Biolamina) coated 3.5 cm^2^ dish and were cultured until confluency in myogenic progenitor proliferation medium without the 1x RevitaCell supplement.

When the sorted myogenic progenitors reach confluency, they can either be cryopreserved, expanded or differentiated towards multinucleated myotubes. The myogenic progenitor proliferation medium was continuously used after FACS sorting, supplemented with 10% DMSO for cryopreservation and replaced by myogenic differentiation medium without supplement for further differentiation towards multinucleated myotubes. Differentiation was started when progenitors reached confluency and harvested after 4 days of differentiation.

### Neuroectoderm differentiation

The protocol for neuroectoderm was adapted from Chetty et al. (2013) and Chambers et al. (2009). hESC were passaged on laminin-521, as described above, 1–2 days prior to neuroectoderm differentiation in a ratio of 50,000–100,000 cells per cm^2^ so that they were 90% confluent on the starting day. The neuroectoderm differentiation medium was refreshed daily and consisted of KnockOut™ DMEM (Thermo Fisher Scientific) with 10% KnockOut™ Serum Replacement (Thermo Fisher Scientific) and supplemented with 500 ng/ml Recombinant Human Noggin Protein (R&D Systems) and 10μM SB431542 (Tocris). Differentiated cells were collected after 12 days of differentiation.

### DNA, RNA extraction and cDNA conversion

DNA was extracted using the DNAeasy Blood & Tissue kit and DNAeasy Micro kit (QIAGEN) and total RNA using the RNAeasy Mini kit (QIAGEN), following the manufacturers' guidelines. RNA was reverse-transcribed to cDNA using the First-Strand cDNA Synthesis Kit (GE Healthcare) following the manufacturers' guidelines.

### qRT-PCR

Expression analysis on myogenic progenitor cells was performed using ViiA7 Real-Time PCR system (Thermo Fisher Scientific) and analysed with VIIA7 software v1.2 (Thermo Fisher Scientific). The 20 µl reaction mix contained 40 ng cDNA, 10 µl TaqMan^®^ Fast Advanced Master Mix (Applied Biosystems) and either 1 µl TaqMan assay (Thermo Fisher Scientific) for *MYOG*, *MYOD1* or 1,8 µM primer mix (IDT) and 250 nM probes (Thermo Fisher Scientific) for *GAPDH* and *UBC* (Table S15). *GAPDH* and *UBC* were used as endogenous controls. Expression analysis on neuroectoderm was similar as above but was performed on 20 ng cDNA input and qPCR Mastermix Plus-low ROX (Eurogentec) was used. The TaqMan assay for *PAX6* was used and *GUSB* and *UBC* (Table S15) were the endogenous controls.

### Immunocytochemistry

Skeletal muscle cells were fixed with 4% paraformaldehyde (PFA) for 10 min at room temperature, washed twice with PBS and permeabilized using 0.3% Triton-X (Sigma-Aldrich) for 10 min. The cells were blocked with PBS with 3% BSA and 0.1% Tween for 30 min, and were incubated with the primary for 1 h, in PBS with 0.1% BSA and 0.1% Tween. The secondary antibody was incubated for 30 min, in PBS with 0.1% BSA and 0.1% Tween. Images were taken by confocal microscopy using an LSM800 (Carl Zeiss). An estimation of the skeletal muscle differentiation efficiency was performed by calculating the ratio of the number of nuclei within a MF20 positive cell on the total amount of nuclei present. For neuroectoderm, the cells were permeabilized with 0.1% Triton (Sigma-Aldrich) and blocked with 10% Fetal Bovine Serum (Thermo Fisher Scientific). The antibodies were diluted in 3% BSA, 0.1% tween, 10% Fetal Bovine Serum in PBS. The primary antibody was incubated overnight at 4°C and the secondary antibody was incubated for 2–3 h at RT. The full list of antibodies and their dilutions can be found in the Table S16.

### Analysis of CTG instability by PacBio Massive Parallel Sequencing

In order to amplify only one to five DNA molecules per reaction, we used a small pool PCR with an input of 20 to 50 pg as described in Seriola et al. (2011). For each cell DNA sample, 20 PCR reactions with low input template DNA were analysed to establish the distribution of the repeat sizes in each sample (De Temmerman et al., 2008; Seriola et al., 2011; Barbé et al., 2017). Repeats were amplified with high fidelity using the LongAmp Taq polymerase (New England Biolabs). Twenty to 50 pg of DNA was amplified in a 25 µl reaction mix containing 2.5 units LongAmp Taq DNA polymerase, 1x LongAmp buffer (New England Biolabs), 0.2 mM dNTPs (Illustra DNA polymerization mix, GE Healthcare) and 0.4 µM of primers DM101 and DM102 (Integrated DNA Technologies) (Brook et al., 1992) and 2.5% dimethyl sulfoxide (DMSO). Primer sequences are listed in Table S17. Amplification conditions were as follows: 4 min of initial denaturation at 94°C, 35 cycles of 30 s denaturation at 94°C, 8 min annealing and extension at 65°C and a final extension step at 65°C for 10 min. The LongAmp amplicons, spanning the repeat, were prepared for sequencing as described in PacBio's guide for Preparing SMRTbell™ Libraries using PacBio^®^ Barcoded Adapters for Multiplex SMRT^®^ Sequencing. This protocol allows to pool two samples in one library that each consist of 20 PCR products with a different barcode. Before exonuclease treatment, 500 ng of PUC19 plasmid was added to avoid degradation of intact SMRTbells. Each library was sequenced on a single SMRT cell by a PacBio RS II or Sequel using the DNA/Polymerase binding Kit P6 v2 (Pacific Biosciences) for a 360 min movie. We used PacBio's DNA Sequencing Reagent Kit 4.0 v2 for all runs. Therefore, demultiplexed circular consensus (CCS) reads were generated with the RS_ReadsOfInsert.1 protocol from PacBio's SMRT portal (v2.3.0) or with ccs and lima software from SMRTLink (v6.0.0) with a minimum of one full pass, a minimum predicted accuracy of 90% and demultiplexing with symmetric barcodes. Next, each PCR product was aligned to the *DMPK* CTG repeat using BWA-SW v0.7.10 (Li and Durbin, 2009) against the human reference genome hg19 downloaded from UCSC (Karolchik et al., 2004), followed by conversion of SAM to BAM by Samtools v1.3.1 (Li et al., 2009). To finally convert to BED format and select the on-target CCS reads BEDtools v2.20.1 was used (Quinlan and Hall, 2010). For each CCS read spanning the CTG repeat, the number of repeat units was determined by measuring the distance between two unique regions flanking the CTG repeat followed by detecting the most abundantly present repeat size in each PCR product, here represented by the median.

### Bisulfite treatment and massive parallel sequencing

Bisulfite treated massive parallel sequencing was performed as described by Barbé et al. (2017). Briefly, the Imprint DNA Modification Kit (Sigma-Aldrich) was used for bisulfite treatment on 200 ng DNA. Bisulfite-treated DNA was amplified using primers in Table S17 for regions upstream (CTCF1) and downstream (CTCF2) of the CTG repeat, using the Jumpstart Taq DNA Polymerase Kit (Sigma-Aldrich). The first and second round PCR conditions were adapted from Barbé et al. (2017). First round PCR primers (Table S17) are indicated by ‘1’ at the end of the target name, second round primers (Table S17) are indicated by Miseq at the end of the target name. Libraries were made as described in Barbé et al. (2017) and subsequently loaded on the MiSeq Reagent Nano Kit v2 (500 cycles) according to the manufacturer's instructions and sequenced at 2×250 bp (Illumina). During data analysis, we used on online tool (https://tabsat.ait.ac.at) that includes all sequences that have been sequenced.

### RNA sequencing and analysis

Total RNA was quality checked using a Fragment Analyzer, and 500 ng was depleted of rRNA (NEBNext rRNA Depletion kit, NEB) and cDNA libraries prepared (NEBNext UltraII Directional RNA library prep kit, NEB). Sequencing was performed on the NextSeq500 Illumina platform using version 2.5 chemistry. Fastq files were inspected using FastQC [FastQC: A Quality Control Tool for High Throughput Sequence Data: http://www.bioinformatics.babraham.ac.uk/projects/fastqc/ (2015)]. Reads were filtered and trimmed to a read length of 70 using BBDuk (sourceforge.net/projects/bbmap). For the RNAseq analysis, Fastq sequences were mapped against the Genome Reference Consortium Human Build 38 (GRCh38.p13). The software used for mapping was STAR (version 2.7.3) (Dobin et al., 2013). The RNA-seq by Expectation Maximization (RSEM) (Li and Dewey, 2011) software (version 1.3.2) was used to produce the count table for each sample. RSEM algorithm was chosen because it is optimized for multi-mapped reads. The RNA-seq analysis was performed using the R software (version 3.6.3) with the edgeR (Robinson et al., 2009) and DESeq2 (Love et al., 2014) libraries. Only genes with a count per million (cpm) greater than 1 in at least two samples were considered. The raw counts were normalized using the trimmed mean of M values (Robinson and Oshlack, 2010) (TMM) algorithm. For each comparison, a different general linear model was built. Statistical testing was done using the empirical Bayes quasi-likelihood *F*-test. The normalized counts were then transformed in a log_2_ fold-change (log_2_FC) table with their associated statistics, *P*-value and false discovery rate (FDR). In each comparison, genes with a |log_2_FC|>1 and an FDR<0.05 were considered as significantly differentially expressed. A |log_2_FC|>1 means at least two times more or two times less transcript in the test group in comparison to the control group. The Gene set enrichment analysis (GSEA) software was downloaded from (http://software.broadinstitute.org/gsea/). The ranking score for each score was computed for each coding gene CPM>1 in at least two samples. The parameters set for each analysis were: enrichment statistic as weighted, number of permutations was 1000, exclude sets larger than 500 and exclude sets smaller than 15. The libraries used from Molecular Signatures Database v6.2 (MSigDB) were hallmark gene sets (H), curated gene sets (C2) and ontology gene sets (C5). The gene sets were statistically relevant if their FDR was below 0.05. The gene sets were considered as positively enriched if their normalized enriched score (NES) was above 1.4 and negatively enriched if their NES<−1.4 (Subramanian et al., 2005).

For splicing analysis, files were aligned to the human genome (hg38) using STAR (v2.6.0a) (Dobin et al., 2013) and splicing was quantified using rMATS (v4.0.2) (Shen et al., 2014). All splicing events were categorised in five different classes of splicing events: cassette or skipped exon (SE), mutually exclusive exon (MXE), alternative 5′ splice site (A5SS), alternative 3′ splice site (A3SS) and retained intron (RI) events. Data were filtered based on average read number ≥5 and FDR ≤0.05 and the percentage spliced-in (Ψ, PSI) was calculated. This dataset was further used to compare non-DM1 with DM1 samples using the change in percent spliced in (ΔΨ) and splicing events with a cut-off of |ΔΨ|>0.1 were further included. We also analysed splicing events over the timecourse of myogenic differentiation based on average read number ≥5 and FDR ≤0.05 and cut-off of |ΔΨ|>0.1.

### Statistics

We used the Jonckheere-Terpstra Test, which is based on comparing medians to study differences in median repeat size across cell lines and conditions. A result of *P*<0.05 in the Jonckheere-Terpstra test indicates that our data follows a specific trend.

## Supplementary Material

Supplementary information
