# Supplementary material for: Myotonic dystrophy type 1 embryonic stem cells show decreased myogenic potential, increased CpG methylation at the DMPK locus and RNA mis-splicing
Source: Biol Open. 2022 Jan 12;11(1):bio058978. doi: 10.1242/bio.058978 (PMC8764412; doi:10.1242/bio.058978)
Supplement: Supplementary information [file biolopen-11-058978-s1.pdf]

Supplemental data

Table of contents

FIG. S1: CTG REPEAT SIZE IN THE FOUR HESCS AND THEIR DERIVED MYOBLASTS AND MYOTUBES. 2
TABLE S1: INDIVIDUAL CTG REPEAT VALUES FOR THE FIG. S1 AND IS PRESENTED IN A SEPARATE EXCEL SHEET. 3
FIG. S2: SAMPLES CLUSTER TOGETHER ACCORDING TO CELL TYPE NOT ACCORDING TO DM1 VS NON-DM1. .. 4
TABLE S2: LIST OF GO PATHWAYS RELATED TO MUSCLE DIFFERENTIATION COMMONLY OR DIFFERENTIALLY REGULATED IN DM1 OR NON-DM1 CELL LINES FROM HUMAN EMBRYONIC STEM CELLS TO MYOBLASTS. 5
TABLE S3: LIST OF GO PATHWAYS RELATED TO MUSCLE DIFFERENTIATION COMMONLY OR DIFFERENTIALLY REGULATED IN DM1 OR NON-DM1 CELL LINES FROM MYOBLASTS TO MYOTUBES. 7
TABLE S4: GENES INCLUDED IN THE HEATMAP IN FIGURE 1E. 9
TABLE S5: GENES INCLUDED IN THE HEATMAP IN FIGURE 2D. 12
FIG. S3. HEATMAPS REPRESENTING THE LOG FOLD CHANGE OF GENES BELONGING TO IL6 JAK STAT3 SIGNALING, AND TNFA SIGNALING VIA NFKB PATHWAYS. 15
TABLE S6: GENES INCLUDED IN THE HEATMAPS IN FIGURE 3B AND FIG. S3. 16
FIG. S4: THE CPG METHYLATION DOWNSTREAM OF THE CTG REPEAT IN DM1 SAMPLES AND NON-DM1 SAMPLES OVER THE COURSE OF DIFFERENTIATION. 31
FIG. S5: CHARACTERIZATION OF THE CELLS AFTER NEUROECTODERM DIFFERENTIATION. 32
FIG. S6: THE UPSTREAM CPG METHYLATION TENDS TO INCREASE OVER NEUROECTODERM DIFFERENTIATION IN DM1 SAMPLES, THE DOWNSTREAM CPG REGION REMAINS UNCHANGED. 33
TABLE S7: SPLICING DEFECTS COMPARING DM1 MYOBLASTS VERSUS MYOTUBES 34
TABLE S8: SPLICING DEFECTS COMPARING DM1 STEM CELLS VERSUS MYOTUBES 34
TABLE S9: SPLICING DEFECTS COMPARING NON-DM1 LINES VERSUS VUB03_DM1 34
TABLE S10: SPLICING DEFECTS COMPARING NON-DM1 MYOTUBES VERSUS DM1 MYOTUBES 34
TABLE S11: SPLICING COMPARING NON-DM1 MYOTUBES VERSUS NON-DM1 MYOTUBES 34
TABLE S12: SPLICING DEFECTS COMPARING NON-DM1 MYOTUBES VERSUS DM1 MYOTUBES 34
TABLE S13: SPLICING DEFECTS COMPARING NON-DM1 STEM CELLS VERSUS DM1 STEM CELLS 34
TABLE S14: SPLICING COMPARING NON-DM1 STEM CELLS VERSUS NON-DM1 MYOTUBES 34
TABLE S15: PROBES, ASSAYS AND PRIMERS FOR QRT-PCR. 34
TABLE S16: LIST OF USED ANTIBODIES AND MANUFACTURERS. 35
TABLE S17: LIST OF PRIMERS. 35

Biology Open • Supplementary information

A

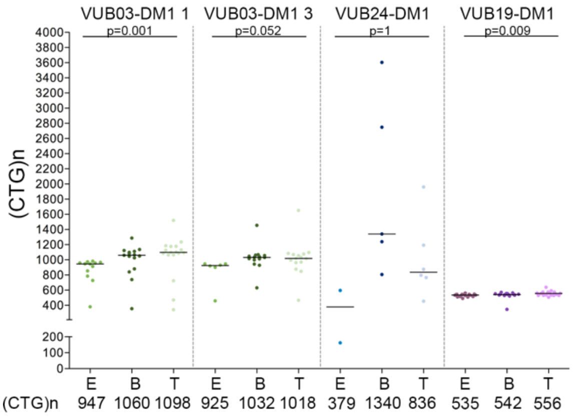

B

Jonckheere-Terpstra Test

VUB03-DM1 1 SC-MB-MT	data	VUB03-DM1 3 SC-MB-MT	data
number of levels	3	number of levels	3
N	43	N	35
Observed J-T Statistic	453	Observed J-T Statistic	255
Mean J-T statistic	303.5	Mean J-T statistic	192
std deviation of J-T statistic	44.631	std deviation of J-T statistic	32.357
std J-T statistic	3.35	std J-T statistic	1.95
Asymp. Sig. (2-tailed)	0.001	Asymp. Sig. (2-tailed)	0.052

VUB24-DM1 SC-MB-MT	data	VUB19-DM1 SC-MB-MT	data
number of levels	3	number of levels	3
N	13	N	54
Observed J-T Statistic	26	Observed J-T Statistic	649
Mean J-T statistic	26	Mean J-T statistic	485.5
std deviation of J-T statistic	7.461	std deviation of J-T statistic	62.97
std J-T statistic	0	std J-T statistic	2.596
Asymp. Sig. (2-tailed)	1	Asymp. Sig. (2-tailed)	0.009

Fig. S1. CTG repeat size in the four hESCs and their derived myoblasts and myotubes.

(A) The median CTG repeat size is shown for all 3 cell types (hESCs, myoblasts, myotubes) of VUB03-DM1, VUB24-DM1 and VUB19-DM1. Every dot represents the median repeat size of one low input LongAmp PCR reaction, and reflects the repeat size which was dominantly present in that PCR reaction. The horizontal line for every dot cluster represent the median for that cluster. Individual repeat sizes of every dot are presented in **Table S1**. **(B)** The Jonkheere-Terpstra test details. The Jonckheere-Terpstra test was performed on every hESC line separately, including the three different cell types. N is the number of data points.

Abbreviations: E and SC: human embryonic stem cells; B and MB: myoblasts; T and MT: myotubes.

Biology Open • Supplementary information

Table S1. Individual CTG repeat values for the Fig. S1 and is presented in a separate excel sheet.

PacBio results are shown per individual LongAmp PCR reaction and per DM1 cell line for hESCs, myoblasts and myotubes. Number of reads, maximum and minimum repeat size, median and mean CTG repeat sizes are shown per PCR reaction. The values at the bottom of the rows with the mean and median per PCR reaction indicate the mean or median for the whole sample.

https://figshare.com/s/491658bfa49266b27e9a

Biology Open • Supplementary information

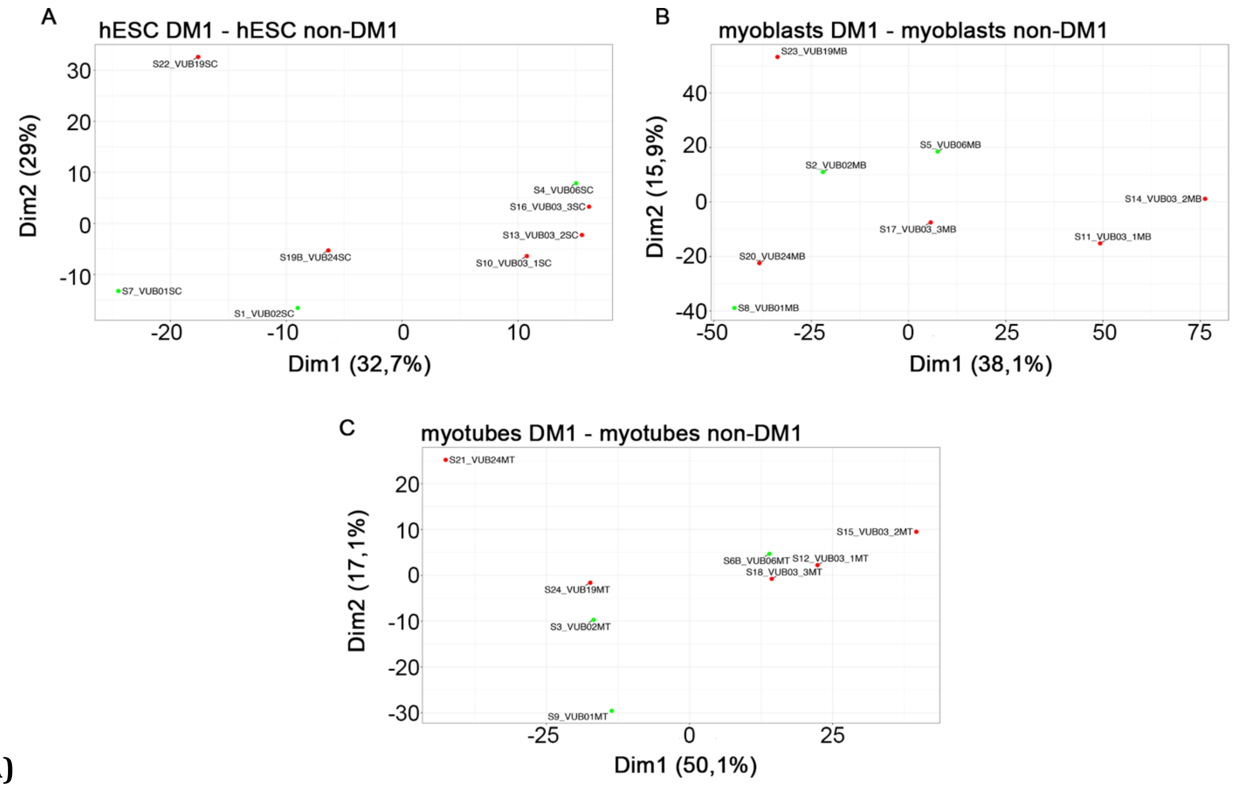

(A)

Fig. S2. Samples cluster together according to cell type not according to DM1 vs non-DM1.

(A) Principle component analysis of DM1 hESCs versus non-DM1 hESCs, **(B)** Principle component analysis of DM1 myoblasts versus non-DM1 myoblasts, **(C)** Principle component analysis of DM1 myotubes versus non-DM1 myotubes. Abbreviations: SC: human embryonic stem cells; MB: myoblasts; MT: myotubes.

Biology Open • Supplementary information

Table S2. List of GO pathways related to muscle differentiation commonly or differentially regulated in DM1 or non-DM1 cell lines from human embryonic stem cells to myoblasts.

Abbreviations: NES: normalized enrichment score

common in DM1 and nonDM1		
**pathway**	**padj**	**NES**
GO_ACTIN_FILAMENT_BASED_PROCESS	0.01265662	1.82755139
GO_ACTIN_FILAMENT_BINDING	0.01265662	1.65965972
GO_ACTIN_FILAMENT_BUNDLE	0.01265662	1.72006638
GO_ACTIN_FILAMENT_ORGANIZATION	0.01265662	1.80606287
GO_ACTIN_MONOMER_BINDING	0.01265662	1.92364276
GO_ACTOMYOSIN	0.01265662	1.92669322
GO_MESODERM_DEVELOPMENT	0.02718075	1.51100596
GO_MESODERM_MORPHOGENESIS	0.01265662	2.09475924
GO_MESODERMAL_CELL_DIFFERENTIATION	0.02718075	1.79060856
GO_MUSCLE_CELL_DEVELOPMENT	0.03352309	1.53029422
GO_MUSCLE_CELL_DIFFERENTIATION	0.01265662	2.00327926
GO_MUSCLE_CELL_MIGRATION	0.01265662	2.0880279
GO_MUSCLE_STRUCTURE_DEVELOPMENT	0.01265662	2.14812435
GO_MUSCLE_TISSUE_DEVELOPMENT	0.01265662	2.11831806
GO_MYOBLAST_DIFFERENTIATION	0.01265662	1.91262265
GO_MYOBLAST_FUSION	0.01265662	1.97777147
GO_POSITIVE_REGULATION_OF_MUSCLE_CELL_DIFFERENTIATION	0.01265662	1.88645592
GO_POSITIVE_REGULATION_OF_MYOBLAST_DIFFERENTIATION	0.01265662	1.95881106
GO_POSITIVE_REGULATION_OF_MYOTUBE_DIFFERENTIATION	0.02718075	1.70879793
GO_POSITIVE_REGULATION_OF_SKELETAL_MUSCLE_TISSUE_DEVELOPMENT	0.02047867	1.74902239
GO_POSITIVE_REGULATION_OF_SMOOTH_MUSCLE_CELL_MIGRATION	0.01265662	1.9709965
GO_POSITIVE_REGULATION_OF_STRIATED_MUSCLE_CELL_DIFFERENTIATION	0.01265662	1.75918725
GO_REGULATION_OF_ACTIN_FILAMENT_BASED_PROCESS	0.01265662	1.71984935

Biology Open • Supplementary information

GO_REGULATION_OF_ACTIN_FILAMENT_DEPOLYMERIZATION	0.01265662	1.76999533
GO_REGULATION_OF_ACTIN_FILAMENT_LENGTH	0.01265662	1.87975112
GO_REGULATION_OF_MYOBLAST_DIFFERENTIATION	0.01265662	1.96570676
GO_REGULATION_OF_SKELETAL_MUSCLE_TISSUE_DEVELOPMENT	0.01265662	1.92787985
GO_REGULATION_OF_SMOOTH_MUSCLE_CELL_PROLIFERATION	0.01265662	1.64533319
GO_SKELETAL_MUSCLE_CELL_DIFFERENTIATION	0.01265662	1.8912858
GO_SKELETAL_MUSCLE_ORGAN_DEVELOPMENT	0.01265662	1.85674546
GO_SMOOTH_MUSCLE_CELL_DIFFERENTIATION	0.02047867	1.72172324
GO_SMOOTH_MUSCLE_TISSUE_DEVELOPMENT	0.01265662	2.10317899
**only in nonDM1**		
**pathway**	**padj**	**NES**
GO_MUSCLE_ORGAN_MORPHOGENESIS	0.02063181	1.79042048
GO_RESPONSE_TO_MUSCLE_STRETCH	0.0271632	1.72427596
GO_NEGATIVE_REGULATION_OF_MUSCLE_TISSUE_DEVELOPMENT	0.0391335	1.6233145
GO_POSITIVE_REGULATION_OF_MUSCLE_TISSUE_DEVELOPMENT	0.0391335	1.60740162
**only in DM1**		
**pathway**	**padj**	**NES**
GO_MUSCLE_CELL_PROLIFERATION	0.01265662	1.9935163
GO_MUSCLE_CELL_CELLULAR_HOMEOSTASIS	0.02047867	1.92889912
GO_STRUCTURAL_CONSTITUENT_OF_MUSCLE	0.01265662	1.87357571
GO_MYOFILAMENT	0.02718075	1.75936398
GO_MYOFIBRIL_ASSEMBLY	0.02047867	1.70005522
GO_ACTIN_MYOSIN_FILAMENT_SLIDING	0.04057227	1.66507122
GO_POSITIVE_REGULATION_OF_SMOOTH_MUSCLE_CELL_PROLIFERATION	0.04630565	1.56071946

Biology Open • Supplementary information

Table S3. List of GO pathways related to muscle differentiation commonly or differentially regulated in DM1 or non-DM1 cell lines from myoblasts to myotubes.

Abbreviations: NES: normalized enrichment score

common in DM1 and nonDM1		
**pathway**	**padj**	**NES**
GO_ACTIN_FILAMENT_BASED_MOVEMENT	0.0182555	1.84590055
GO_ACTIN_MEDIATED_CELL_CONTRACTION	0.0181814	2.19563341
GO_ACTIN_MYOSIN_FILAMENT_SLIDING	0.0181814	2.17862925
GO_ACTION_POTENTIAL	0.0182555	1.80776418
GO_ACTOMYOSIN_STRUCTURE_ORGANIZATION	0.02939984	1.61938644
GO_MUSCLE_CELL_DEVELOPMENT	0.01858137	1.68050193
GO_MUSCLE_CELL_DIFFERENTIATION	0.01985494	1.63851458
GO_MUSCLE_CONTRACTION	0.01960765	1.8034354
GO_MUSCLE_ORGAN_DEVELOPMENT	0.02014791	1.74404638
GO_MUSCLE_STRUCTURE_DEVELOPMENT	0.02126039	1.68710183
GO_MUSCLE_SYSTEM_PROCESS	0.01959137	1.80356905
GO_MUSCLE_TISSUE_DEVELOPMENT	0.031786	1.53735186
GO_MYOFIBRIL_ASSEMBLY	0.0181814	1.99425034
GO_MYOFILAMENT	0.0181814	1.7783661
GO_MYOSIN_II_COMPLEX	0.0181814	1.85943125
GO_REGULATION_OF_MUSCLE_SYSTEM_PROCESS	0.0313491	1.38870282
GO_SKELETAL_MUSCLE_ORGAN_DEVELOPMENT	0.01858137	1.60400751
GO_STRIATED_MUSCLE_CELL_DIFFERENTIATION	0.0313491	1.54769956
GO_STRIATED_MUSCLE_CONTRACTION	0.04783482	1.51151411
GO_STRUCTURAL_CONSTITUENT_OF_MUSCLE	0.0181814	2.07551241
**only in nonDM1**		
**pathway**	**padj**	**NES**
GO_ACTIN_FILAMENT_BUNDLE_ORGANIZATION	0.02500129	-1.7082806
GO_MESODERM_MORPHOGENESIS	0.03669841	-1.5419403
GO_POSITIVE_REGULATION_OF_ACTIN_FILAMENT_POLYMERIZATION	0.01180616	-1.8566163
GO_REGULATION_OF_ACTIN_FILAMENT_LENGTH	0.01180616	-1.6299536
GO_ACTIN_FILAMENT_BASED_PROCESS	0.01180616	1.52952967
GO_ACTININ_BINDING	0.04629302	1.63068784
GO_MUSCLE_FIBER_DEVELOPMENT	0.01180616	1.78597005
GO_MUSCLE_HYPERTROPHY	0.01180616	2.02477825

Biology Open • Supplementary information

GO_MYOSIN_COMPLEX	0.01180616	1.76727751
GO_MYOSIN_FILAMENT	0.01180616	2.10635835
GO_POSITIVE_REGULATION_OF_SKELETAL_MUSCLE_TISSUE_DEVELOPMENT	0.042022	1.6967193
GO_REGULATION_OF_ACTIN_FILAMENT_BASED_MOVEMENT	0.02468595	1.76604176
GO_REGULATION_OF_MUSCLE_ADAPTATION	0.01180616	1.75761499
GO_REGULATION_OF_MUSCLE_CELL_DIFFERENTIATION	0.04185	1.41027052
GO_REGULATION_OF_MUSCLE_CONTRACTION	0.01180616	1.826582
GO_REGULATION_OF_MUSCLE_TISSUE_DEVELOPMENT	0.01916667	1.60113238
GO_REGULATION_OF_SKELETAL_MUSCLE_TISSUE_DEVELOPMENT	0.04496701	1.54391244
GO_REGULATION_OF_STRIATED_MUSCLE_CONTRACTION	0.01180616	1.84471796
GO_SKELETAL_MUSCLE_CELL_DIFFERENTIATION	0.01180616	2.02984286
GO_SKELETAL_MUSCLE_CONTRACTION	0.01180616	1.99822543
GO_STRIATED_MUSCLE_ADAPTATION	0.02468595	1.88461344
GO_ALPHA_ACTININ_BINDING	0.04619133	1.64472534

**only in DM1**		
**pathway**	**padj**	**NES**
GO_MUSCLE_MYOSIN_COMPLEX	0.0181814	2.03464647

Biology Open • Supplementary information

Table S4. Genes included in the heatmap in Figure 1E.

This heatmap includes the differential gene expression from hESC to Myoblast for the genes involved in the development of skeletal muscle, as listed in the Hallmark Myogenesis library of GSEA. The table and heatmap only include values for genes with an FDR<0.05. FC: log2 fold change, FDR: false discovery rate.

	Non DM1		DM1	
**Gene**	**Log2FC**	**FDR**	**Log2FC**	**FDR**
PGAM2	-7.6364474	0.00050996	-4.1372501	0.00228038
ACTN3	-7.1062215	0.00169109	-4.9963056	0.00141402
VIPR1	-6.684418	0.00011729	-6.5755281	1.1054E-05
GNAO1	-6.1910983	4.1641E-05	-6.0808602	1.0679E-07
ACTA1	-5.3121312	0.00459045		
KCNH2	-5.1236904	0.00183986	-2.6026062	0.01207283
MYH2	-5.0635294	0.00520164	-2.0813847	0.02477974
FXYD1	-4.8936168	0.00169415		
PTGIS	-4.5349603	0.00461408	-3.3097771	0.00055805
SORBS1	-4.204472	5.6523E-05	-3.193996	1.3774E-06
ERBB3	-3.8512355	0.00043801		
HRC	-3.6297947	0.01160065		
MYOZ1	-3.482335	0.00717859	-2.6183037	0.02848058
CACNA1H	-3.411508	0.00461981	-2.6953797	0.00109621
PYGM	-3.3522263	0.01775395		
GJA5	-3.0896497	0.00474154	-1.8749708	0.04047632
CKB	-2.8788679	0.00326882		
FGF2	-2.5262915	0.00955584	-3.2596399	0.00104467
CLU	-2.3593071	0.01343509	-1.3162698	0.0061474
SOD3	-2.2542128	0.02673005		
PTP4A3	-2.1849369	0.00643007	-1.1815027	0.04094168
SLC6A8	-2.0690044	0.0002861	-1.6167478	1.3472E-05
FKBP1B	-2.0080864	0.0059866	-2.0506157	5.2646E-06
SH3BGR	-1.8581192	0.01436419	-1.7977259	0.00025212
GPX3	-1.8481428	0.03745818	-2.3940478	0.00012671
FOXO4	-1.826665	0.0068506	-1.6908778	0.00308057
CFD	-1.7590589	0.03498819	-2.2272838	0.00151544
LARGE1	-1.3101683	0.00102905	-0.9173283	0.00416748
NOS1	-1.2629876	0.02469434		
LAMA2	-1.2051618	0.02739755		
HDAC5	-1.1630103	0.00354764	-0.8288821	0.00606047
ABLIM1	-1.1609756	0.0013776		
MYH11	-1.1157403	0.03127347		
FABP3	-1.0708169	0.04014091		
OCEL1	-0.9854368	0.02040891	-0.942291	0.00134744
DTNA	-0.9735365	0.01792914	-0.7680321	0.02640046

Biology Open • Supplementary information

AGL	-0.6869365	0.03243653		
PFKM	-0.4816099	0.03997276		
SCD	-0.4237087	0.04357237		
BAG1	0.52291356	0.03661361		
TPM3	0.5531979	0.02759006		
SH2B1	0.63269704	0.01812807	0.65558606	0.00456142
SPTAN1	0.6420548	0.02553393	0.8783119	0.00133428
GABARAPL2	0.76464736	0.00303357	0.79314506	0.0011554
ITGB5	0.84040954	0.0433597	0.64552128	0.00101551
DMPK	0.93892448	0.03801914	2.33839218	0.00481972
CNN3	0.95240685	0.00765872	0.8327663	0.00028287
SORBS3	0.96911095	0.00389133	0.70863702	0.00207322
MEF2A	1.03887422	0.00189322	1.67415001	0.00167855
HSPB8	1.06612798	0.0461395		
MAPK12	1.1287699	0.00696582	0.71548276	0.00924735
MEF2D	1.21509755	0.00035714	1.45068928	0.00044345
CHRNB1	1.28042522	0.0279838	2.13804309	0.01104284
PKIA	1.30821332	0.00351101	1.57559488	6.2214E-05
MRAS	1.35782071	0.00285817	2.66211884	0.00114645
PLXNB2	1.42669337	0.00073714	1.30189246	1.2604E-05
RB1	1.50245783	0.00088596	1.37371906	6.5545E-05
CRAT	1.54450558	0.00122494	1.81393102	1.8562E-05
ACSL1	1.54936383	0.03491145	1.66672016	0.00069319
PPP1R3C	1.63281955	0.03958486	1.77194249	0.00447141
MYO1C	1.7080987	0.00099124	1.25387419	2.4556E-05
KLF5	1.74023751	0.00602375	2.91415737	0.00127289
GADD45B	1.74119308	0.00469833	1.49723194	0.01308782
AK1	1.78885198	0.00087464	1.99424536	0.00022705
BDKRB2	1.82705386	0.03652561	2.40704318	0.00141779
STC2	1.84520704	0.01910589	2.09760189	0.01258574
BIN1	1.88045433	0.00025973	2.67073101	0.0012023
AEBP1	1.88665576	0.00077659	1.4746003	0.00698089
IGFBP3	1.91990724	0.01449659	2.58270264	0.00037647
LPIN1	2.04999279	0.00011903	2.00339165	8.1572E-06
NCAM1	2.25890788	0.00478228	3.93859662	0.00028913
NQO1	2.30884621	0.00069818	2.07424127	0.00019916
SGCA	2.37896995	0.02383693	4.62772958	0.01354487
SIRT2	2.45797159	0.00028937	3.5285191	0.00035307
MYH3	2.50843605	0.02332573	6.95308417	0.00703814
PDLIM7	2.72154165	7.5496E-05	2.64355517	1.7823E-06
TPM2	2.80871992	0.00078469	2.93465456	8.1347E-06
MYLK	2.83151781	0.00443069	2.76635452	1.8483E-05
CTF1	2.87495473	0.00077659	2.47213046	0.00018835

Biology Open • Supplementary information

MYH9	2.94513142	0.00015746	2.49330023	4.8667E-06
SVIL	3.01375511	0.00048534	2.95019569	7.4847E-08
COL4A2	3.04453224	0.00090295	2.78856241	5.8933E-05
TAGLN	3.06344378	0.00107551	1.87855956	0.00392026
ITGB1	3.0831708	0.00017315	2.60753692	7.4703E-06
GSN	3.09622146	0.0006844	3.52197322	3.2212E-05
SPARC	3.13473222	0.00019542	2.77060242	8.6243E-06
COL6A2	3.13819281	0.00077529	2.44537108	1.3031E-06
COL15A1	3.16355938	0.01475994	5.30371238	0.00108984
COL1A1	3.18915404	0.00104841	3.20779302	0.00025212
EPHB3	3.20310055	0.01904683	4.43428353	0.00073895
SMTN	3.27096114	0.00020537	2.63538877	1.0895E-05
MYLPF	3.31537411	0.01292004	7.21912669	0.00660378
SSPN	3.49300238	0.00029642	4.18483126	7.3464E-05
TGFB1	3.61486094	0.00163264	3.59273433	2.0195E-06
WWTR1	3.67345661	5.9126E-05	3.61272202	1.0047E-06
MYBPH	3.77887647	0.02689955	9.05159429	0.00545013
PRNP	3.96818459	0.00032957	3.45581746	1.0663E-05
KCNH1	3.97792156	0.00032767	4.612112	1.0941E-05
MEF2C	4.23754976	0.00011903	7.50750712	0.00047727
KIFC3	4.46366974	0.00106394	4.0230589	4.1261E-07
SPHK1	4.50026891	0.00011983	3.76449516	0.00044345
CDKN1A	4.51078872	0.00093463	3.45718467	0.00192736
HBEGF	4.53786655	0.00278658	3.48187621	0.00141428
LSP1	4.83654505	0.00989165	5.39217829	0.00094403
BHLHE40	4.86953754	0.00055626	3.84464037	2.5057E-05
MYOG	5.60690782	0.01467014	9.67484023	0.00227968
COL6A3	6.37157841	0.00048213	6.10605019	1.3089E-07
ADAM12	6.50025487	0.00017569	6.86283676	2.0486E-07
CDH13	6.52977417	0.00034417	6.0534943	1.6907E-06
SGCD	6.60504238	9.2577E-05	8.31569881	0.00036429
IGFBP7	7.86491015	0.00040475	7.40743712	1.35E-06
COL3A1	8.18258098	0.0037429	8.7579506	3.229E-06
MYL4	9.10699754	0.00855629	7.5034369	0.00370619
CHRNA1	9.43800137	9.337E-05	10.3710038	0.00016395

Biology Open • Supplementary information

Table S5. Genes included in the heatmap in Figure 2D.

This heatmap includes the differential gene expression from Myoblast to Myotube for the genes involved in the development of skeletal muscle, as listed in the Hallmark Myogenesis library of GSEA. The table and heatmap only include values for genes with an FDR<0.05. FC: log2 fold change, FDR: false discovery rate.

	Non DM1		DM1	
**Gene**	**Log2FC**	**FDR**	**Log2FC**	**FDR**
LAMA2	3.434466542	0.004964119	2.558175579	0.002277686
COL3A1	2.969467392	0.042640266	2.955398545	0.003068552
DMD	2.767419811	0.009233199	2.785701929	0.003537978
SORBS1	3.356207514	0.010852453	2.837099843	0.004705953
IGF1	7.961961829	0.021266006	6.601104416	0.005055313
ABLIM1	2.032069952	0.007201063	1.303012396	0.006641053
GJA5	7.350950766	0.002335051	5.903106902	0.011650128
TSC2	0.588021565	0.022460709	0.513039783	0.011790923
ITGB5	-1.029597026	0.026519573	-1.095285801	0.011876489
CRYAB	4.338175471	0.034820232	4.160120198	0.012695223
KCNH2	6.514672136	0.005838995	4.339128578	0.012740782
FABP3	3.917470453	0.018246247	2.844873071	0.014690511
NQO1	-1.585215391	0.011538682	-1.701832158	0.016059109
SSPN	2.325759034	0.008582274	1.854418399	0.016346349
HSPB2	7.605691691	0.007093457	5.701880592	0.016384447
AGL	2.19265002	0.010213886	2.202678156	0.01644433
TGFB1	-1.4248959	0.01054286	-1.499853944	0.016523106
CKMT2	5.510813574	0.007047628	4.359050833	0.019705103
CD36	6.080426272	0.00316201	4.824471292	0.019878718
TCAP	3.558564383	0.032768447	3.69826496	0.021562269
ACHE	2.587647573	0.012991014	2.852586981	0.023164772
IGFBP3	2.158494815	0.038977168	2.034892733	0.024755399
SGCG	5.431689296	0.008587939	3.852361426	0.025090027
SPTAN1	0.891044981	0.026626872	1.02850752	0.025130091
MYL6B	2.271145922	0.005809485	2.011751398	0.025749356
FST	2.397183778	0.027555253	2.57317549	0.02608245
NAV2	0.932944466	0.043370045	1.452889679	0.02704499
FOXO4	2.35168128	0.01166004	2.003659929	0.027181795
PSEN2	2.464107869	0.010811335	2.214830878	0.02744485
SVIL	0.90121126	0.047568627	0.983028954	0.029391722
SPHK1	-2.711792186	0.014526251	-2.363562269	0.033438218
SLN	6.689599383	0.020011912	5.239655295	0.034400081
STC2	-1.878642266	0.021167168	-1.401806338	0.034646023
TNNC2	9.265908784	0.003587066	5.654457864	0.036055991
CACNG1	12.8305577	0.002038081	5.851439514	0.036566693
KLF5	3.317568511	0.005502216	2.267084435	0.036849485

Biology Open • Supplementary information

MAPRE3	2.703345609	0.008206323	1.800803074	0.037299158
EIF4A2	0.538257268	0.015001838	0.523123553	0.03787702
COX6A2	7.460777533	0.026418018	4.278824389	0.038200662
PKIA	1.719028816	0.011076449	1.523168325	0.038819486
LDB3	6.076404982	0.004860348	3.719347159	0.039278995
SORBS3	-1.001467484	0.02041369	-0.550195828	0.041575078
SPEG	1.56457793	0.047736298	1.266826569	0.042457936
CAMK2B	3.697435383	0.021131387	2.620925713	0.04257657
TPM3	-1.116836487	0.007923713	-0.806040209	0.043181949
PRNP	-0.958295281	0.043240439	-0.911032826	0.04653724
CSRP3	5.176373525	0.015988941	3.822859929	0.049102734
MYL1	8.690728906	0.005249948	4.997857263	
ITGB1	-1.32925361	0.012021653	-0.956007405	
MYOM1	8.406506963	0.00437319	4.64701646	
MYOM2	6.149523336	0.009035857	3.951858259	
FXYD1	5.844446055	0.015558367	3.8650738	
TNNI2	8.721228713	0.00383752	4.527387147	
MRAS	2.93920966	0.002788993	1.814355448	
PFKM	0.53224965	0.029222214	0.651726107	
MYH7	7.370984742	0.010366123	4.306603954	
NCAM1	3.693855646	0.008582274	2.021400055	
DTNA	1.390336879	0.008571628	1.230408857	
DES	7.034624445	0.004698949	3.991609377	
CASQ2	8.629082168	0.005614601	4.269249435	
REEP1	4.211069025	0.021167168	2.801647477	
TNNT3	9.110154294	0.006431548	4.377343417	
MYL4	6.992537157	0.006402841	3.650003756	
MYH3	9.621307216	0.006432159	4.201878419	
ATP2A1	4.708730253	0.016644763	3.365619922	
ERBB3	5.219661863	0.003163749	2.760341145	
HRC	8.424311718	0.004502011	3.651198099	
CKM	10.24258556	0.004458363	4.58463117	
MEF2C	5.093576545	0.003993906	2.832725766	
ACTA1	8.031125266	0.002788993	3.735337947	
PTP4A3	1.812624089	0.038026673	1.23971904	
PC	1.488430115	0.039138238	1.195889434	
MYLPF	8.283638421	0.00437319	3.739768021	
CHRNG	5.137513774	0.007488854	2.445679116	
ACTN2	7.826633701	0.003473675	3.651630753	
MYH9	-1.273563434	0.016945518	-0.851558215	
MAPK12	-0.482987897	0.033912329	-0.528816105	
CAV3	7.610257638	0.003427943	3.225843372	
RYR1	6.04432954	0.014192291	3.044566438	

BIN1	1.795943956	0.008310537	1.383843173
SGCD	3.14043155	0.00437319	2.166361257
SGCA	4.713782962	0.00494004	2.798394202
SIRT2	1.737372248	0.006447267	1.332688915
PPFIA4	3.497203613	0.01497645	1.830181151
ENO3	4.48638605	0.013809202	2.260413772
LPIN1	-0.542167579	0.019825178	-0.382264166
MYH8	10.04747762	0.007033353	3.832054535
COL15A1	4.448857219	0.006614878	1.525656537
TNNT1	3.566188914	0.031091592	1.998472503
TNNT2	5.315065188	0.026132879	2.616352844
DMPK	1.680564158	0.005490022	1.129093845
TNNC1	7.914029201	0.002599395	2.770542097
ACTC1	6.249879394	0.011480179	2.758916827
TNNI1	6.175935646	0.016910263	2.458983265
CKB	3.613770376	0.008629059	1.788170616
MYBPH	8.177178805	0.009033672	2.769047414
CHRNB1	1.533927426	0.03392957	1.168247921
MYOG	6.047326723	0.018906755	2.385511018
MEF2D	0.522128005	0.015614808	0.391114879
SMTN	-0.985283507	0.03666777	-0.18946943

Biology Open • Supplementary information

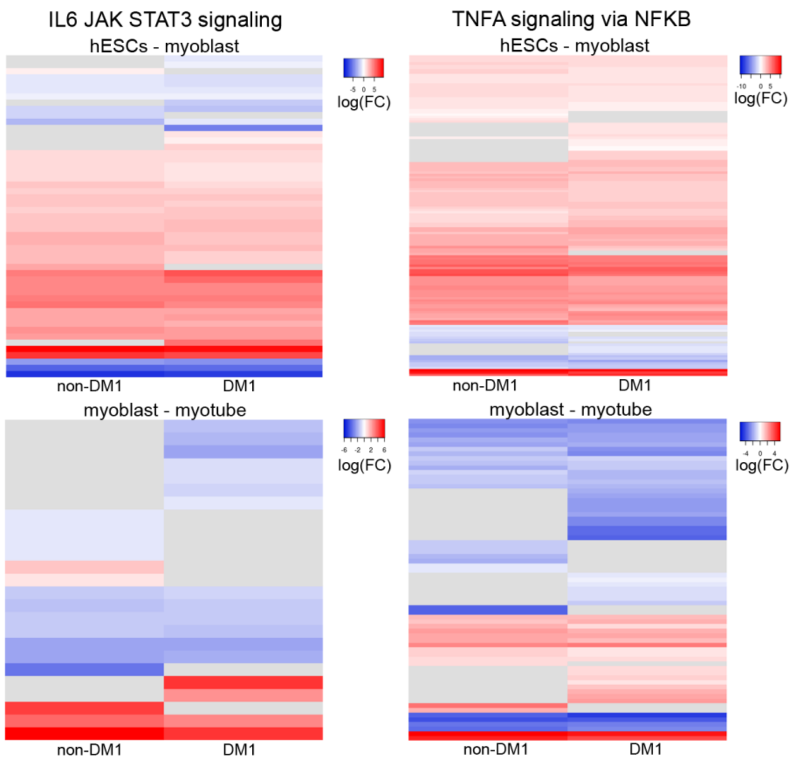

Fig. S3. Heatmaps representing the log fold change of genes belonging to IL6 JAK STAT3 signaling, and TNFA signaling via NFKB pathways.

Grey lines indicate genes with FDR>0.05. Abbreviation: NES: normalized enrichment score; FC: fold change.

Biology Open • Supplementary information

Table S6. Genes included in the heatmaps in Figure 3B and Fig. S3.

Genes in the heatmap including the differential gene expression from stem cell to myoblast for the genes involved in the interferon alfa response, as listed in the Hallmark Interferon Alfa Response library of GSEA. The table and heatmap only include values for genes with an FDR<0.05. FC: log2 fold change, FDR: false discovery rate.

	Non DM1		DM1	
**Gene**	**Log2FC**	**FDR**	**Log2FC**	**FDR**
RTP4	-9.8710063	0.00293264	-9.1145961	6.7332E-06
BST2	-5.6689928	0.00255575	-3.711485	0.00012479
C1S	-3.6145526	0.00392271	-3.0921577	0.00013651
IFITM1	-3.783782	0.00072326	-2.6419353	9.8759E-05
DHX58	-2.6405175	0.00193729	-2.6029477	0.00179974
BATF2	-3.673551	0.0026175	-2.3881501	0.00069974
IFI30	-2.6087761	0.00148313	-2.033113	0.00088395
MX1			-1.2735789	0.01345829
OAS1	-0.9674436	0.00665148	-1.1838165	0.00029696
CD74	-1.9637571	0.00468261	-1.1216428	0.0034503
CCRL2			-0.9619376	0.03405482
TRIM14	-1.0517142	0.01177066	-0.838942	0.00210212
ADAR	-0.4538553	0.04326151	-0.5490263	0.02580798
PNPT1			-0.5315989	0.00552356
NUB1	0.52746854	0.0226403	0.32253053	0.01901764
CMTR1	0.31552143	0.0556944	0.35378361	0.00860065
LAP3	0.5442846	0.01821382	0.39143634	0.01018341
SLC25A28	0.66935904	0.03862344	0.50214236	0.00673699
OGFR			0.54021449	0.00991492
HLA-C			0.63838056	0.04410707
PARP12	0.89486011	0.01799961	0.69650345	0.00106813
TRIM5	0.86991497	0.00539115	0.69817181	0.01045006
TRIM25	0.72164805	0.00253848	0.76537427	0.00103142
NCOA7			0.8193937	0.0001103
CNP	0.91720551	0.00075643	0.93802208	9.2195E-05
TDRD7	0.97659574	0.03256812	0.98949348	0.00395936
MOV10			0.99001671	0.00089653
PARP14			1.03639898	0.00016168
IFIH1			1.07481521	0.04631453
PSME2	1.03072437	0.00182484	1.16555153	0.00026052
CASP8	1.68294852	0.00618372	1.18602132	0.00456825
TAP1	1.02090412	0.01523837	1.21394533	0.00147742
ELF1	0.95741278	0.03258727	1.3127996	4.2495E-06
IRF2	1.2394834	0.00328061	1.35173854	0.000708
STAT2	1.31630803	0.00361701	1.48633341	1.6408E-05
PSME1	1.04515988	0.00389609	1.52652453	0.00044223

Biology Open • Supplementary information

CD47	1.69648427	0.00107538	1.60142131	0.00011415
LAMP3	1.90135588	0.00602392	1.60559618	9.7329E-05
EPSTI1			1.64657152	0.00390111
IL4R	1.69524039	0.00088818	1.65641998	5.886E-05
LGALS3BP	1.81575474	0.00880171	1.84981875	0.00021108
LPAR6	-2.1040376	0.00247152	1.8846873	0.00049018
DDX60	1.21449143	0.02815044	1.92595807	0.0004263
PSMB8	1.45747843	0.00678832	1.97870711	7.9371E-05
SP110	1.90136616	0.00273328	2.01496921	3.2379E-06
OASL			2.02315681	0.00013915
CMPK2	1.69994872	0.02669673	2.13970382	0.00022211
IFI35	1.66901342	0.02967756	2.29758269	0.00054942
IRF9	1.95929158	0.00064738	2.40028118	1.0354E-05
IL15	2.77078982	0.01500403	2.47188007	2.5073E-05
PSMB9			2.48831084	0.0018983
PARP9	2.17773954	0.00223631	2.82072021	1.4696E-05
RSAD2			2.87013131	0.00081155
CSF1	3.27242899	0.00057967	2.89144771	9.9354E-05
TENT5A	3.28138347	0.00159613	3.39027052	1.0692E-05
CXCL10	4.05629843	0.01356744	3.45304179	0.00127289
CXCL11	4.05629843	0.01356744	3.45304179	0.00127289
IRF1	3.11318206	0.00071388	3.51620023	2.9883E-07
B2M	3.41653854	0.00029518	3.79500874	4.6206E-06
IL7	4.26428611	4.1641E-05	4.14466523	3.2372E-07
IFIT3	3.44806214	0.00396568	4.14775246	2.8538E-06
IFIT2	3.24152066	0.01379642	4.24433006	4.4988E-06
GMPR	2.95956246	0.0048119	4.43908047	7.0662E-05
SAMD9	5.00413302	0.00100066	5.33463933	9.1734E-05
IFI27	4.68177109	0.00025745	5.34058862	8.8438E-07
UBA7	4.7775481	0.00262178	6.19323937	4.1436E-06
IFI44L	7.74855836	0.00460375	7.66551219	5.7307E-05
SAMD9L	8.38014829	0.00112005	7.69210855	2.9942E-05
GBP2	6.29738047	0.00556511	8.21587074	1.6461E-05
GBP4	8.02976632	4.1641E-05	8.87883336	3.3853E-08
IFI44	10.4740058	0.00215472	10.6670916	6.1887E-05
SELL	-1.086175	0.00286981		
TRIM21	0.84855874	0.01841346		
USP18	1.68356502	0.01309787		

Biology Open • Supplementary information

Genes in the heatmap including the differential gene expression from myoblast to myotube for the genes involved in the interferon alfa response, as listed in the Hallmark Interferon Alfa Response library of GSEA. The table and heatmap only include values for genes with an FDR<0.05. FC: log2 fold change, FDR: false discovery rate.

	Non DM1		DM1	
**Gene**	**Log2FC**	**FDR**	**Log2FC**	**FDR**
TRIM25	-1.0161334	0.00407008	-1.2705931	0.00127928
PSMA3	-0.5968589	0.01195124	-0.6457465	0.00145744
CD47	-0.8292759	0.01368849	-0.8668103	0.03670892
GMPR	2.0767409	0.01461171		
USP18	1.02658003	0.0431299		
TRIM21	-0.5586301	0.04649472		
IL15			-1.2110939	0.00660677
IRF1			-2.2835597	0.00754558
PARP12			-1.0440708	0.00886797
CASP8			-0.8250892	0.00955576
PSME2			-0.9304172	0.01070195
IL4R			-0.8773788	0.01497536
OGFR			-0.5435969	0.02437785
IFI35			-1.6916088	0.02590576
PSMB8			-1.3500904	0.02745125
IFIT3			-1.4702349	0.0295467
MVB12A			-0.3519241	0.0317893
PLSCR1			-0.8445372	0.03327363
LGALS3BP			-1.3929268	0.03612207
EPSTI1			1.54308177	0.03908688
SP110			-0.6331772	0.04014417
CMPK2			-1.0556658	0.04543234
C1S			2.65304697	0.04649819
MOV10			-0.6852287	0.0495637

Biology Open • Supplementary information

Genes in the heatmap including the differential gene expression from myoblast to myotube for the genes involved in the mTORC1 signaling, as listed in the Hallmark mTORC1 - Genes up-regulated through activation of mTORC1 complex- library of GSEA. The table and heatmap only include values for genes with an FDR<0.05. FC: log2 fold change, FDR: false discovery rate.

	Non DM1		DM1	
**Genes**	**Log2FC**	**FDR**	**Log2FC**	**FDR**
GGA2	1.39395718	0.00390196		
ERO1A	-1.3435659	0.00418508	-1.474942	0.0015409
CD9	-1.3961864	0.00437319		
ENO1	-1.6348037	0.0056941	-1.677491	0.00096568
EDEM1	-1.1586112	0.00640284		
EGLN3	5.14064024	0.00640284		
GAPDH	-1.2550856	0.00644727		
VLDLR	1.20587612	0.00663228		
AURKA	-1.7566402	0.00667111	-1.7725795	0.00289283
LDHA	-2.0607803	0.00744482	-2.1336723	0.0015409
TXNRD1	-0.7779018	0.00765844		
SRD5A1	-1.129161	0.00831054		
NUP205	-0.7874342	0.00831054		
SLC2A1	-1.7736938	0.00858794		
TFRC	-1.7180821	0.00874769	-1.3687902	0.00145744
SLC9A3R1	-1.034294	0.00900165		
GBE1	-1.2782428	0.00952393		
RRM2	-1.6230648	0.01001437	-1.8757533	0.00354383
PSMA3	-0.5968589	0.01195124	-0.6457465	0.00145744
FGL2	3.1913025	0.01259737		
BUB1	-1.7073953	0.01281787	-2.2026589	0.00129302
PLOD2	-1.3974453	0.01290488		
ACTR2	-0.7889837	0.01293796		
IDH1	-0.5842155	0.01375289		
BTG2	1.32461259	0.01461171		
PPIA	-0.7345603	0.01461171		
POLR3G	-1.3856651	0.01473787		
PNP	-2.150089	0.0157362		
EBP	-0.9706267	0.01614303		
DDX39A	-0.8585984	0.01625575		
MCM4	-0.9702213	0.01827781		
PSMD12	-0.6742016	0.01914544		
PSMD14	-0.7025409	0.01961858		
PSMC2	-0.4749448	0.0197571		
ACTR3	-0.8638794	0.02035067		
SLC6A6	-1.5638856	0.02065512		
PSME3	-0.5587381	0.02083138		

Biology Open • Supplementary information

ACLY	-0.6157864	0.02083138		
TPI1	-0.7977651	0.02314094		
RPA1	-0.6340378	0.02484571	-0.7649368	0.00384886
FDXR	-0.903531	0.02518585		
MAP2K3	-1.334313	0.02557279	-1.1479065	0.00314594
CCT6A	-0.5660775	0.02613288		
MCM2	-1.0112654	0.02698042		
GLA	-1.1385406	0.02843371		
PSMD13	-0.5794091	0.02853326		
PSMG1	-0.6666482	0.02879422		
ADIPOR2	-0.4734363	0.0290815		
DHFR	-0.9707819	0.02926847		
PPA1	-0.7761836	0.0321806		
TUBG1	-0.9423582	0.03292739		
GSR	-0.7397882	0.0345282	-0.9562429	0.00246353
CCNF	-0.8882368	0.03460799	-1.3355966	0.00156703
GPI	-0.5971081	0.0347557		
ATP6V1D	-0.5788874	0.03599709		
CACYBP	-0.6562446	0.03629176		
UNG	-0.6598043	0.03632507		
PLK1	-1.8293413	0.03674075	-2.1954151	0.00204983
SORD	-0.510324	0.03801432		
INSIG1	-1.2541102	0.0389763		
GLRX	-0.9993069	0.03945555		
SLA	4.00791479	0.04056427		
CYP51A1	0.58406845	0.04186838	0.84543848	0.00175946
HSPE1	-0.6618585	0.04619077		
ETF1	-0.5282685	0.04631241		
CYB5B	-0.4931647	0.04649472		
AK4	-1.2301485	0.04649472		
CDC25A	-0.8583768	0.04848467		
EEF1E1	-0.6903684	0.04991954		
NAMPT			-1.2050141	0.00396463

Biology Open • Supplementary information

Genes in the heatmap including the differential gene expression from myoblast to myotube for the genes involved in the canonical WNT signaling, as listed in the Hallmark WNT beta catenin signaling (Genes up-regulated by activation of WNT signaling through accumulation of beta catenin CTNNB1) library of GSEA. The table and heatmap only include values for genes with an FDR<0.05. FC: log2 fold change, FDR: false discovery rate.

	Non DM1		DM1	
**Gene**	**Log2FC**	**FDR**	**Log2FC**	**FDR**
DLL1	3.02490657	0.00357914		
PSEN2	2.46410787	0.01081134	2.21483088	0.02744485
NKD1	4.15693784	0.01546835	2.71488895	0.01281312
AXIN2	2.25386868	0.0160369	1.65954167	0.00394503
LEF1	2.33688891	0.01660013		
DKK1	-3.5601985	0.01701892		
SKP2	1.40983119	0.0191727		
WNT5B	-2.184473	0.02528114		
KAT2A	0.48746473	0.02801512		
JAG2	1.80956951	0.03902908		
GNAI1			0.4562088	0.02530757
TP53			-0.3964804	0.03756924
NUMB			-0.4112431	0.04817564

Genes in the heatmap including the differential gene expression from stem cell to myoblast for the genes involved in the IL6-JAK-STAT3 signaling, as listed in the Hallmark IL6-JAK-STAT3 signaling (Genes up-regulated by IL6 [GeneID=3569] via STAT3 [GeneID=6774], e.g., during acute phase response) library of GSEA. The table and heatmap only include values for genes with an FDR<0.05. FC: log2 fold change, FDR: false discovery rate.

	Non DM1		DM1	
**Gene**	**Log2FC**	**FDR**	**Log2FC**	**FDR**
IL6ST	4.26428611	4.1641E-05	4.14466523	3.2372E-07
OSMR	4.50750983	4.4035E-05	4.64246571	1.6061E-07
CD44	7.1533028	5.6523E-05	6.32644941	1.0077E-07
ITGA4	4.75482789	9.337E-05	5.98071831	3.5189E-07
PDGFC	5.0575846	9.8128E-05	4.33901364	1.0601E-07
IFNAR1	1.97760937	9.8973E-05	1.92092762	4.7556E-07
STAM2	1.92235282	0.00017136	1.83483037	5.6503E-07
PIK3R5	-7.9610759	0.00020256	-7.7903413	5.7319E-06
JUN	2.69348552	0.00021404	2.02752476	0.00287086
TYK2	1.80504095	0.00026724	1.86369814	9.7896E-08
IL1R1	4.41550369	0.00029969	5.28856073	5.135E-06
TNFRSF12A	2.33354197	0.00030757	1.71676477	0.00063839
ITGB3	9.29609163	0.00034365	8.69967984	1.0041E-05
CSF1	3.27242899	0.00057967	2.89144771	9.9354E-05
PTPN1	1.13157544	0.00061288	0.88953856	0.00071451
IRF9	1.95929158	0.00064738	2.40028118	1.0354E-05
IL10RB	2.54786608	0.00069408	2.35882848	1.5019E-05
IRF1	3.11318206	0.00071388	3.51620023	2.9883E-07
IL4R	1.69524039	0.00088818	1.65641998	5.886E-05
INHBE	-6.1033354	0.00111856	-5.6992855	0.00109621
SOCS3	1.88891489	0.00120972	2.59961308	0.00022229
TNFRSF1A	2.01843338	0.00144618	2.14672085	2.0286E-06
LEPR	1.23861536	0.00155782	1.23600981	0.0031101
TGFB1	3.61486094	0.00163264	3.59273433	2.0195E-06
IFNGR2	1.1425538	0.00211175	0.76948797	0.00090272
FAS	2.49815015	0.00237573	1.82850815	0.00316376
IL17RA	1.97799425	0.00313285	1.45886921	1.7864E-05
STAT2	1.31630803	0.00361701	1.48633341	1.6408E-05
CBL	-0.8496222	0.00384032	-1.0970443	5.335E-05
PTPN2	-0.7237833	0.0057656	-0.7274825	0.00011301
IL13RA1	3.12011256	0.00614545	3.26150887	9.4861E-07
CNTFR	-3.7908074	0.00658418	-4.4382957	0.00077272
TNFRSF1B	-2.9195845	0.00874089	-1.0890794	0.03153736
PIM1	-0.9760434	0.00962678	-1.3917178	0.00263493
TLR2	2.75568692	0.01338685	2.07934841	0.00118947
CXCL1	4.05629843	0.01356744	3.45304179	0.00127289

CXCL3	-1.5796414	0.01695678	-2.3876273	0.00531439
LTB	4.21479792	0.01856928	4.16486875	0.00101537
LTBR	-1.7181058	0.02377898		
IL17RB	-0.9959749	0.03588731	-1.2219837	0.00261062
PTPN11	0.45438024	0.04112286		
STAT1	1.26168843	0.04498209	1.01012492	0.00016363
TNFRSF21			0.71504354	0.00097337
BAK1			-0.62338	0.00460355
CSF2RA			-2.0893389	0.00515515
A2M			5.17346952	0.00537684
MYD88			0.86705336	0.01076212
CD9			-0.7455295	0.01546379

Biology Open • Supplementary information

Genes in the heatmap including the differential gene expression from myoblast to myotube for the genes involved in the IL6-JAK-STAT3 signaling, as listed in the Hallmark IL6-JAK-STAT3 signaling (Genes up-regulated by IL6 [GeneID=3569] via STAT3 [GeneID=6774], e.g., during acute phase response) library of GSEA. The table and heatmap only include values for genes with an FDR<0.05. FC: log2 fold change, FDR: false discovery rate.

	Non DM1		DM1	
**Gene**	**Log2FC**	**FDR**	**Log2FC**	**FDR**
CD44	-2.431373	0.00233505	-2.1887476	0.00332717
CD36	6.08042627	0.00316201	4.82447129	0.01987872
CD9	-1.3961864	0.00437319	-1.3202866	0.00490647
TGFB1	-1.4248959	0.01054286	-1.4998539	0.01652311
PTPN1	-0.5990345	0.01400909		
CBL	-0.5498962	0.02630241		
HMOX1	-1.4525025	0.0280504	-1.010035	0.04107062
INHBE	3.42769504	0.03045296	2.91778296	0.01524444
A2M	4.59053439	0.03329534		
IL6ST	-0.6436064	0.03440211		
LEPR	-0.7105066	0.03478801		
TNFRSF12A	-1.6472052	0.03877374	-1.2299748	0.04556517
ITGA4	1.29781713	0.03945555		
STAT3	0.66499655	0.04262235		
ITGB3	-2.2464195	0.04905305	-2.4031601	0.04865699
ACVRL1			2.5921333	0.00238614
IL17RA			-0.8701443	0.00391008
IRF1			-2.2835597	0.00754558
TNFRSF21			-0.6900455	0.0103455
IL4R			-0.8773788	0.01497536
TNFRSF1A			-1.0208199	0.03128259

Biology Open • Supplementary information

Genes in the heatmap including the differential gene expression from stem cell to myoblast for the genes involved in the TNFA signaling via NFKB, as listed in the Hallmark TNFA signaling via NFKB (Genes regulated by NF-kB in response to TNF [GeneID=7124].) library of GSEA. The table and heatmap only include values for genes with an FDR<0.05. FC: log2 fold change, FDR: false discovery rate.

	Non DM1		DM1	
**Gene**	**Log2FC**	**FDR**	**Log2FC**	**FDR**
IL6ST	4.26428611	4.1641E-05	4.14466523	3.2372E-07
PLAU	5.88192357	4.1641E-05	5.54780578	1.9938E-06
CD44	7.1533028	5.6523E-05	6.32644941	1.0077E-07
LIF	4.49126759	8.1067E-05	2.90652773	0.00127148
FOSL2	5.60709275	8.9611E-05	5.15794233	3.3853E-08
RELA	1.95923029	0.00010117	1.81292324	1.4451E-07
SPHK1	4.50026891	0.00011983	3.76449516	0.00044345
SERPINB8	4.668887	0.00013613	4.1179032	9.738E-07
SMAD3	3.01940803	0.00015057	3.22507329	3.5407E-07
TNC	7.19549432	0.00017773	6.53578839	7.8018E-07
B4GALT1	2.34716809	0.00018416	2.29763423	5.1062E-07
KLF2	5.86774385	0.00021367	3.44550138	0.00489474
CLCF1	4.95462355	0.00021385	4.50462716	3.0993E-05
JUN	2.69348552	0.00021404	2.02752476	0.00287086
IER3	4.61430028	0.00021548	4.16879302	7.4331E-06
GADD45A	2.37615749	0.00022834	1.77177622	0.00586905
INHBA	5.90445041	0.00025618	5.4155506	0.00139389
SPSB1	2.67608397	0.00025973	2.5137276	1.1426E-06
EGR2	-3.567657	0.00026586	-2.3488436	0.00229201
CCND1	3.62372926	0.00027331	1.99576619	0.00036472
DUSP4	4.99023601	0.00028991	6.56130903	1.3912E-05
PLAUR	4.37883786	0.00032423	3.75679352	4.6629E-05
PFKFB3	1.64385068	0.00040511	1.82839884	2.2004E-06
MAFF	3.04888309	0.00042883	2.47066841	0.00046907
TANK	1.47074501	0.0004501	1.51923592	2.3746E-06
PDLIM5	1.56274579	0.00053927	1.35287403	0.00049762
BTG3	1.34733034	0.0005397	0.93795034	0.0002928
BHLHE40	4.86953754	0.00055626	3.84464037	2.5057E-05
NR4A1	-3.5040709	0.00056571	-2.8485536	0.00020533
CSF1	3.27242899	0.00057967	2.89144771	9.9354E-05
FJX1	2.82362985	0.0006258	1.95049402	2.9199E-05
LDLR	1.40863979	0.00067307	0.93166494	0.00474641
PHLDA1	3.15350847	0.00068365	3.00703719	2.1122E-05
TIPARP	2.65146779	0.00068652	2.77018488	2.7692E-06
PLK2	4.18741038	0.00070396	4.16711182	3.1759E-06
IRF1	3.11318206	0.00071388	3.51620023	2.9883E-07
SERPINE1	7.33181838	0.00072915	5.88437571	0.00082336

Biology Open • Supplementary information

SNN	-1.3941582	0.00075036	-1.2038017	8.5995E-05
PTX3	6.60548669	0.00081759	5.42035491	1.9576E-06
PTGS2	3.23038972	0.00081858	2.97712131	0.02046786
EHD1	2.15142068	0.00082951	1.8946288	4.3527E-05
FOSL1	5.48607244	0.00088633	3.8891711	0.00082335
CDKN1A	4.51078872	0.00093463	3.45718467	0.00192736
TNFAIP3	3.35810143	0.00095142	3.27851577	7.3386E-05
IER5	3.03155773	0.0009862	2.30612188	1.0126E-05
KLF6	2.59934461	0.00105975	2.33854264	1.8973E-05
RCAN1	3.84643829	0.00107611	3.13346514	0.00115921
BIRC3	4.56988422	0.00109641	4.81220036	4.2402E-05
PHLDA2	4.59568862	0.00114657	4.34233954	5.8592E-06
TGIF1	-1.2380713	0.00120375	-0.8684406	0.00210157
SOCS3	1.88891489	0.00120972	2.59961308	0.00022229
TNIP1	2.51333976	0.00121207	2.14218623	1.9077E-06
KLF10	1.53785774	0.00128274	1.43648325	0.00111541
LAMB3	4.56557751	0.00147924	2.17779783	0.00883152
NFKB1	2.91851395	0.00149282	2.20798068	9.4429E-07
PTGER4	2.98924487	0.0018416	3.64082741	2.8794E-05
BIRC2	1.19462475	0.00185609	0.96830537	9.1092E-05
LITAF	-2.1644054	0.00195738	-2.8840659	1.2199E-05
IFNGR2	1.1425538	0.00211175	0.76948797	0.00090272
MSC	3.58561922	0.00246516	4.85471623	1.9163E-05
TNFRSF9	9.73702815	0.00259861	7.74972642	2.2307E-06
KLF9	2.54511022	0.00262398	3.5627719	8.8011E-05
PMEPA1	2.34737165	0.00266264	2.0197655	0.00074986
ATP2B1	1.24963088	0.00268751	1.98921288	0.00381694
DUSP2	-3.3430403	0.00277126	-3.8870379	1.3395E-05
HBEGF	4.53786655	0.00278658	3.48187621	0.00141428
CEBPD	1.98691503	0.00282013	2.17173985	0.00025627
AREG	5.378497	0.00297827	5.40119768	8.2429E-05
BCL3	3.88778452	0.00318883	4.43261698	2.3507E-06
JUNB	2.49081017	0.0033155	2.99464106	4.8681E-07
NFIL3	1.3318753	0.00361713	1.14509689	4.451E-05
RHOB	1.99315782	0.00392704	1.25866895	0.02448467
MARCKS	1.4017005	0.00445957	1.27343242	6.8011E-06
GADD45B	1.74119308	0.00469833	1.49723194	0.01308782
IL7R	6.87695247	0.0047553	8.76481041	7.3636E-05
HES1	1.6312793	0.00484687	2.2504904	2.9199E-05
DENND5A	0.7961755	0.00494793	0.64816417	0.00124312
G0S2	3.73971023	0.00504929		
ICOSLG	-1.4611358	0.00516786		
IL1B	3.17661945	0.00604946		

Biology Open • Supplementary information

DUSP1	2.85932912	0.00639778	2.66007103	0.00044989
DNAJB4	1.66752872	0.00646698	1.23238173	0.00204993
SLC2A3	-2.490632	0.00671404	-2.4354326	8.9969E-06
CCL2	3.52144505	0.00721261	4.27255331	6.1722E-05
DUSP5	2.04034973	0.00858352	1.13728097	0.01513775
VEGFA	2.34009207	0.00964687	2.04636415	0.00904804
SDC4	1.27702129	0.00983729		
PER1	-0.9487304	0.01014042	-0.5395033	0.01491049
NFE2L2	0.88435854	0.0105677	1.10888878	0.00011561
NFKBIA	1.12103951	0.01200224	0.74911493	0.008937
RELB	1.46356803	0.0120915	0.97011518	0.00947672
DRAM1	2.26758363	0.01284686	1.7168651	0.00818384
SQSTM1	1.19398864	0.01312401	1.23781692	0.00105948
TLR2	2.75568692	0.01338685	2.07934841	0.00118947
CXCL1	4.05629843	0.01356744	3.45304179	0.00127289
BTG2	1.63590986	0.01361685	1.50688623	0.0119254
IFIT2	3.24152066	0.01379642	4.24433006	4.4988E-06
GCH1	-1.9681908	0.01384497	-1.4944872	0.00673605
PPP1R15A	1.17026443	0.01386943	1.14940237	0.00805603
NFAT5	0.57650618	0.0146681	0.99085339	1.3572E-05
TAP1	1.02090412	0.01523837	1.21394533	0.00147742
CXCL3	-1.5796414	0.01695678	-2.3876273	0.00531439
SERPINB2	10.6075033	0.01786321	9.81919692	0.00049908
MAP2K3	0.94836477	0.01820229	0.80021588	0.00079607
IRS2	0.66352452	0.0193025		
GFPT2	-2.1920174	0.02142384	-1.0625651	0.02509286
TNFAIP8	1.54523973	0.02259913	0.99360976	0.01111727
SGK1	1.35289701	0.02295154	1.99890101	0.0004821
PTPRE	1.53770403	0.02818597	2.54723116	0.00010847
NFKB2	0.89019259	0.02965579		
SOD2	0.39954455	0.03543663		
MXD1	1.22740039	0.03689353	1.088996	0.01695058
EGR3	-2.5848334	0.04389306		
SIK1	1.8359086	0.04523988	2.79649665	0.01681219
EGR1	-1.4830135	0.04526919		
GEM	1.40099197	0.04571177	2.23244479	7.5245E-05
EIF1	0.47845916	0.04798672		
KDM6B			1.78397311	0.00029758
SAT1			1.19718371	0.00030512
PDE4B			-1.7775686	0.0005334
BTG1			1.89339298	0.00088351
TUBB2A			-0.866104	0.00658129
ZC3H12A			1.10377688	0.00917347

ICAM1	1.7576845	0.00997947
CD83	-1.1433299	0.01071153
TRIB1	-1.2813529	0.01164771
SLC2A6	0.66936924	0.0125713
TSC22D1	0.36656198	0.01316564
F3	1.34449689	0.02047407
ABCA1	1.11670153	0.02159121
PLPP3	-0.9462838	0.0289258
DDX58	0.63964827	0.02983679
CCNL1	0.30104418	0.03352216
CFLAR	1.144527	0.03471332
ZFP36	0.77305938	0.03667679
IFIH1	1.07481521	0.04631453

Biology Open • Supplementary information

Genes in the heatmap including the differential gene expression from myoblast to myotube for the genes involved in the TNFA signaling via NFKB, as listed in the Hallmark TNFA signaling via NFKB (Genes regulated by NF-kB in response to TNF [GeneID=7124].) library of GSEA. The table and heatmap only include values for genes with an FDR<0.05. FC: log2 fold change, FDR: false discovery rate.

	Non DM1		DM1	
**Gene**	**Log2FC**	**FDR**	**Log2FC**	**FDR**
PLAU	-3.7253126	0.00233505	-4.4394853	0.00125701
CD44	-2.431373	0.00233505	-2.1887476	0.00332717
CCND1	-3.3758515	0.00256561	-2.854504	0.00652244
KDM6B	1.68461199	0.00278899	0.83548083	0.01987352
ICOSLG	3.06923682	0.00316375		
CEBPD	1.94083309	0.0045315	1.3928021	0.00705977
IL1B	-3.6097374	0.00472061	-3.4161109	0.03199129
EGR2	5.52392201	0.00500721	4.88603149	0.00088082
IER3	-1.9627713	0.00643155		
SERPINB8	-2.0400797	0.00644727	-1.8445558	0.01123286
PLAUR	-3.2580499	0.00728704	-3.0088861	0.00459787
PFKFB3	-1.2937208	0.00774095	-1.5452674	0.00185298
FOSL1	-4.1635059	0.00782501	-3.9771904	0.00085706
SPSB1	-1.2978482	0.00889246		
PER1	0.93214356	0.00941864	0.64116797	0.02365818
ATP2B1	1.71999824	0.01161296		
ZBTB10	0.99628161	0.01183729	0.65840089	0.02068093
PHLDA2	-2.2567037	0.01220746	-2.3755433	0.0089609
ETS2	-0.977517	0.01231359	-1.5892265	0.00404488
VEGFA	-2.5958891	0.01331465	-2.7326907	0.0098362
SPHK1	-2.7117922	0.01452625	-2.3635623	0.03343822
BTG2	1.32461259	0.01461171	1.57064958	0.01837678
MSC	2.13075635	0.0198375	1.73099421	0.0266424
KLF9	-1.7512155	0.0200161	-2.7091698	0.00120837
IRS2	-1.297685	0.02116717	-1.0150547	0.01926929
MAP2K3	-1.334313	0.02557279	-1.1479065	0.00314594
EGR1	2.4955646	0.02913575	2.70121992	0.0043867
PHLDA1	-1.1342375	0.02995247	-1.7543035	0.00278674
DENND5A	-0.5718393	0.03011756		
TNC	1.32976083	0.03011893	1.34942421	0.03199123
LIF	-1.8109141	0.03025927	-2.0833327	0.02430623
EGR3	4.79107308	0.03266606	3.77781214	0.00320201
SAT1	-1.4657417	0.03412257	-1.6196244	0.02090836
IL6ST	-0.6436064	0.03440211		
SNN	0.70054093	0.0362185	0.68996913	0.04236406
BCL6	0.75612844	0.03683152		
ID2	-1.1352427	0.03709973		

Biology Open • Supplementary information

SERPINE1	-3.6072948	0.03771482		
MAFF	-1.8013523	0.03798174	-1.2231861	0.04833985
SDC4	-1.2388338	0.04039827		
INHBA	-1.5555937	0.04132073		
DUSP4	-1.1872072	0.04208609	-2.4941912	0.00384886
NR4A1	1.92101262	0.04405169	1.87336331	0.01163682
G0S2			-3.5975766	0.00363902
NAMPT			-1.2050141	0.00396463
IRF1			-2.2835597	0.00754558
DUSP2			2.13306166	0.00955576
FJX1			0.73479925	0.01074221
ABCA1			-2.1740011	0.01153193
BCL3			-1.9951603	0.01227016
TNIP1			-0.7465809	0.0151081
B4GALT5			-0.61299	0.01746993
AREG			-3.4324499	0.01751988
SQSTM1			-0.8575789	0.01933262
LAMB3			-2.2413078	0.02108977
SLC2A6			-0.8473012	0.02108977
NR4A3			1.86056543	0.02689223
KLF2			1.22503553	0.02836673
TRIP10			-0.3918087	0.0300163
TNF			1.34942421	0.03199123
TUBB2A			0.78802077	0.03437131
F2RL1			-2.8280786	0.03625759
NFAT5			-0.4541473	0.03787702
GFPT2			-2.2206588	0.04171543
CDKN1A			1.68918886	0.04173948
RHOB			0.52301439	0.04269738
PDLIM5			0.90131606	0.04302633

Biology Open • Supplementary information

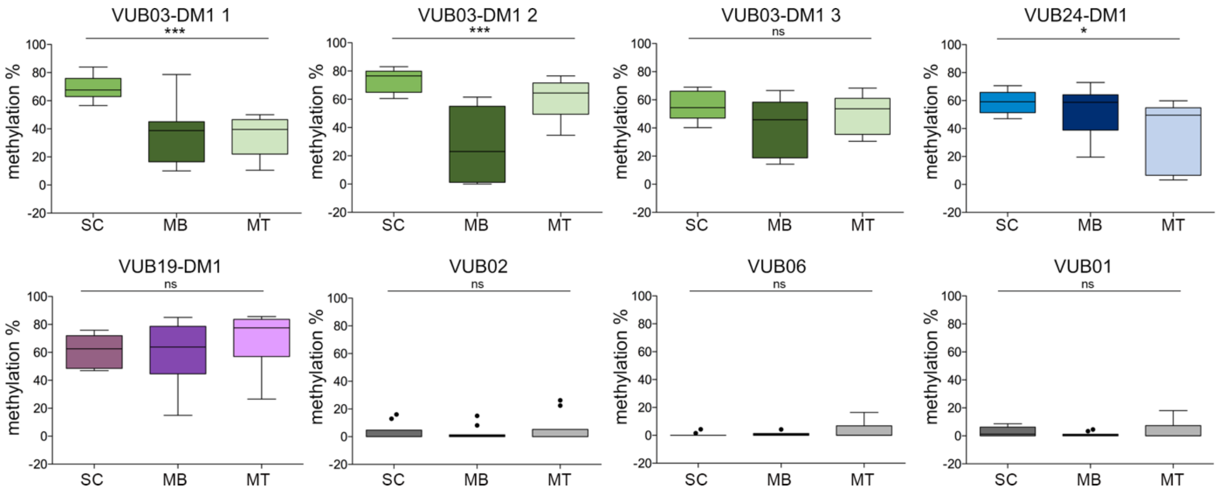

Fig. S4. The CpG methylation downstream of the CTG repeat in DM1 samples and non-DM1 samples over the course of differentiation.

The downstream methylation is shown for 11 CpG sites after massive parallel sequencing for 3 DM1 and 3 non-DM1 cell lines. The methylation status of the downstream region decreases for some lines (One-way Anova VUB03-DM1 1 and 2 $p<0.0001$, VUB24-DM1 $p= 0.0214$) other lines remain unchanged (One-way Anova VUB03-DM1 3 $p=0.0913$, VUB19-DM1 $p=0.4399$, VUB02 $p=0.5037$, VUB06 $p=0.0753$, VUB01 $p=0.2927$). Abbreviations: SC: hESC; MB: myoblasts; MT: myotubes

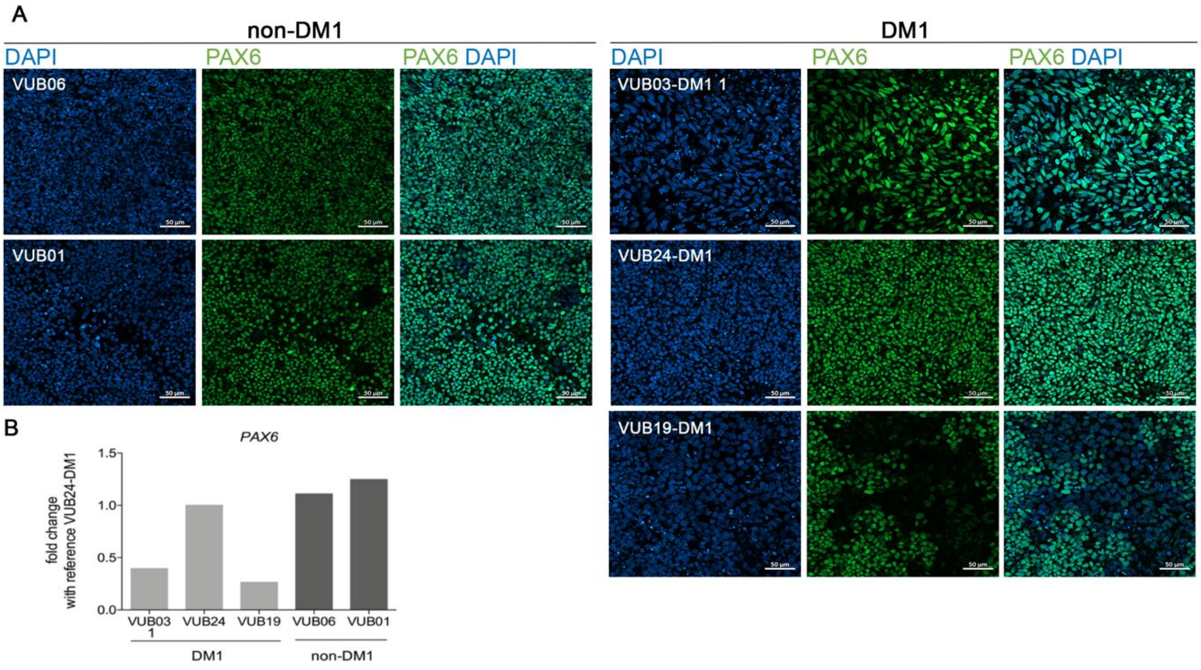

Fig. S5. Characterization of the cells after neuroectoderm differentiation.

(A) Immunostaining for the neuroectoderm marker PAX6 for 2 control non-DM1 cell lines and 3 DM1 cell lines after 12 days of neuroectoderm differentiation. **(C)** *PAX6* mRNA expression neuroectoderm cells relative to the DM1 reference sample VUB24. *UBC* and *GUSB* gene expression was used for normalization. The experiment was only performed once.

Biology Open • Supplementary information

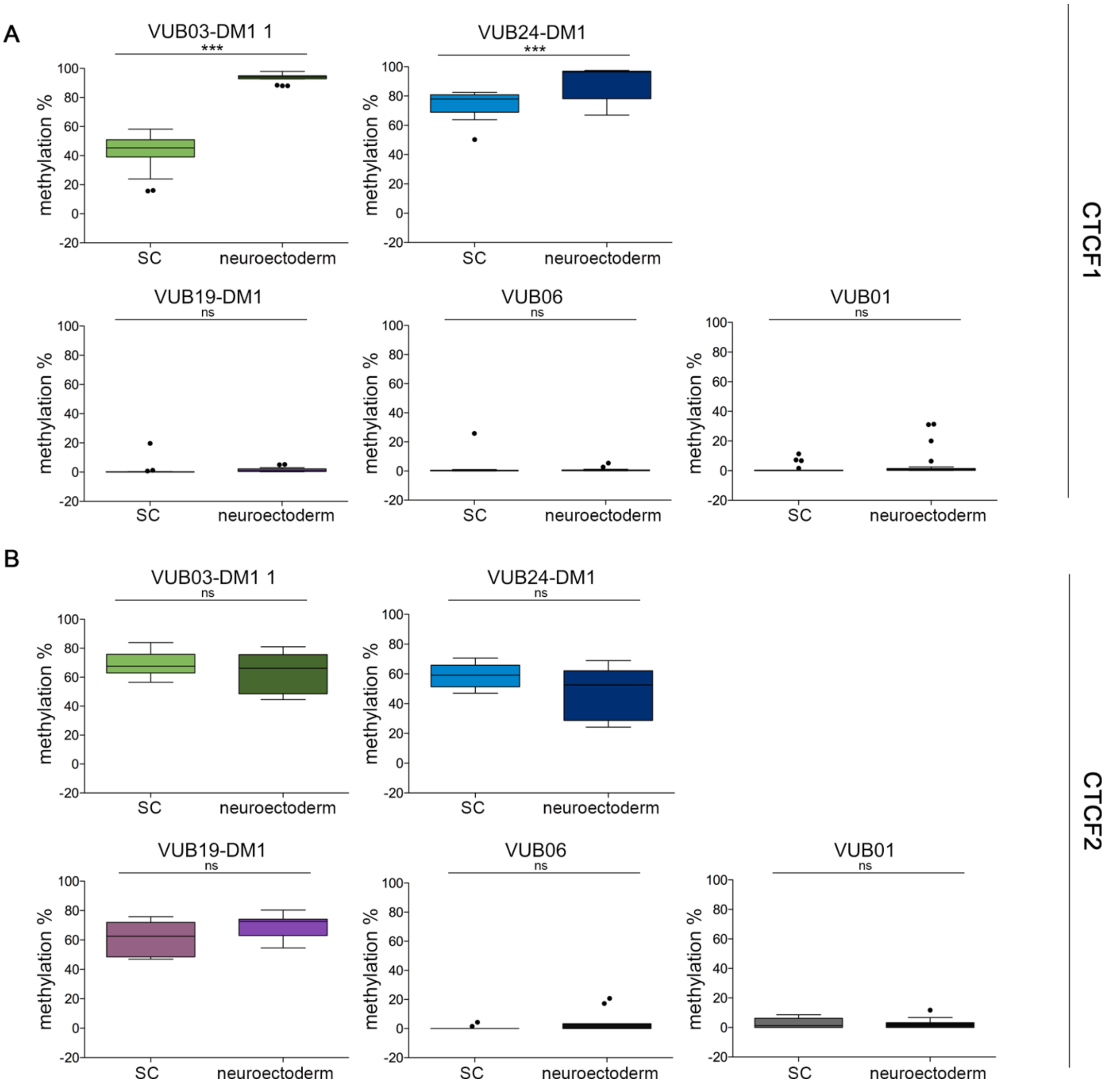

Fig. S6. The upstream CpG methylation tends to increase over neuroectoderm differentiation in DM1 samples, the downstream CpG region remains unchanged.

(A) Methylation levels of the CpG sites upstream of the CTG repeat. The upstream methylation is shown for 23 CpG sites and all epi-alleles were analyzed after massive parallel sequencing for 3 DM1 cell lines and 2 non-DM1 cell lines (one-way Anova). **(B)** Methylation levels of the CpG sites downstream of the CTG repeat. The upstream methylation is shown for 23 CpG sites and all epi-alleles were analyzed after massive parallel sequencing for 3 DM1 cell lines and 2 non-DM1 cell lines (one-way Anova).

Biology Open • Supplementary information

Table S7. Splicing defects comparing DM1 myoblasts versus myotubes
https://figshare.com/s/491658bfa49266b27e9a

Table S8. Splicing defects comparing DM1 stem cells versus myotubes
https://figshare.com/s/491658bfa49266b27e9a

Table S9.: Splicing defects comparing non-DM1 lines versus VUB03_DM1
https://figshare.com/s/491658bfa49266b27e9a

Table S10.: Splicing defects comparing non-DM1 myotubes versus DM1 myotubes
https://figshare.com/s/491658bfa49266b27e9a

Table S11. Splicing comparing non-DM1 myotubes versus non-DM1 myotubes
https://figshare.com/s/491658bfa49266b27e9a

Table S12. Splicing defects comparing non-DM1 myotubes versus DM1 myotubes
https://figshare.com/s/491658bfa49266b27e9a

Table S13.: Splicing defects comparing non-DM1 stem cells versus DM1 stem cells
https://figshare.com/s/491658bfa49266b27e9a

Table S14. Splicing comparing non-DM1 stem cells versus non-DM1 myotubes
https://figshare.com/s/491658bfa49266b27e9a

Table S15. Probes, assays and primers for qRT-PCR.

Gene	TaqMan Assay/ Sequence
MYOG	Hs01072232_m1
MYOD1	Hs00159528_m1
IL6	Hs00174131_m1
TRL3	Hs01551079_g1
IRF7	Hs01014809_g1
PAX6	Hs00240871_m1
GUSB	Hs99999908_m1
UBC	Forward 5'-CGCAGCCGGGATTTG-3'<br>Reverse 5'-TCAAGTGACGATCACAGCGA-3'<br>Probe 6-FAM-TCGCAGTTCTTGTTTGTG-MGB
GAPDH	Forward 5’-ATGGAAATCCCATCACCATCTT-3’<br>Reverse 5’-CGCCCCACTTGATTTTGG-3’<br>Probe 6-FAM-CAGCAGCGAGATCC-MGB

Biology Open • Supplementary information

Table S16. List of used antibodies and manufacturers.

Primary antibodies	Species	Application	Company	Cat#
HNK-1-FITC (B3GAT1)	Mouse	FACS	AVIVA SYSTEMS BIOLOGY	OASA02271
C-MET-APC (anti-HGF R/C-met)	Mouse	FACS	R&D systems	FAB3582A
MF20	Mouse	ICC	DSHB	NS
PAX6	Mouse	ICC	Abcam	ab78545
**Secondary antibodies**	**Species**	**Application**	**Company**	**Cat#**
Alexa Fluor 488	Goat-anti-Mouse	ICC	Invitrogen	A11001

Table S17. List of primers.

Primer sequences for the amplification and massive parallel sequencing of CTCF1 and CTCF2. 'F' indicates the forward primers, 'R' the reverse primers. Number '1' following 'F' or 'R' indicate the first round of the nested PCR. For the second PCR round, primers with Miseq at the end of the target name were used.

Target name	Primer sequence
CTCF1 F1	5'-TGTYGTYGTTTTGGGTTGTATTG-3'
CTCF1 R1	5'-CAACATTCCYGACTACAAAAACCCTT-3'
CTCF2 F1	5'-TTYGGTTAGGTTGAGGTTT-3'
CTCF2 R1	5'-TTAACAAAAACAAATTTCCC-3'
CTCF1 F Miseq	5'-TCGTCGGCAGCGTCAGATGTGTATAAGAGACAGGTTGTATTGGGTTGGTGGTTTA-3'
CTCF1 R Miseq	5'- GTCTCGTGGGCTCGGAGATGTGTATAAGAGACAG CTACAAAAACCCTTYGAACCC-3'
CTCF2 F Miseq	5'-TCGTCGGCAGCGTCAGATGTGTATAAGAGACAGTAAATTGTAGGTTTGGGAAG-3'
CTCF2 R Miseq	5'-GTCTCGTGGGCTCGGAGATGTGTATAAGAGACAGTTAACAAAAACAAATTTCCC-3'
DM101	5'-CTTCCCAGGCCTGCAGTTTGCCCATCCA-3'
DM102	5'-GAACGGGGCTCGAAGGGTCCTTGT-3'

Biology Open • Supplementary information
